# Using multiple outcomes in intervention studies for improved trade-off between power and type I errors:   the Adjust NVar approach

**DOI:** 10.12688/f1000research.73520.1

**Published:** 2021-09-30

**Authors:** Dorothy V. M. Bishop

**Affiliations:** 1 Department of Experimental Psychology, University of Oxford, Oxford, Oxon, OX2 6GG, UK

**Keywords:** intervention, methodology, statistics, correlated outcomes, power, familywise error rate, multiple comparisons

## Abstract

Background

The CONSORT guidelines for clinical trials recommend use of a single primary outcome, to guard against the raised risk of false positive findings when multiple measures are considered. It is, however, possible to include a suite of multiple outcomes in an intervention study, while controlling the familywise error rate, if the criterion for rejecting the null hypothesis specifies that N or more of the outcomes reach an agreed level of statistical significance, where N depends on the total number of outcome measures included in the study, and the correlation between them.

Methods

Simulations were run, using a conventional null-hypothesis significance testing approach with alpha set at .05, to explore the case when between 2 and 12 outcome measures are included to compare two groups, with average correlation between measures ranging from zero to .8, and true effect size ranging from 0 to .7. In step 1, a table is created giving the minimum N significant outcomes (MinNSig) that is required for a given set of outcome measures to control the familywise error rate at 5%. In step 2, data are simulated using MinNSig values for each set of correlated outcomes and the resulting proportion of significant results is computed for different sample sizes,correlations, and effect sizes.

Results

The Adjust NVar approach can achieve a more efficient trade-off between power and type I error rate than use of a single outcome when there are three or more moderately intercorrelated outcome variables.

Conclusions

Where it is feasible to have a suite of moderately correlated outcome measures, then this might be a more efficient approach than reliance on a single primary outcome measure in an intervention study. In effect, it builds in an internal replication to the study. This approach can also be used to evaluate published intervention studies.

## The case against multiple outcomes

The CONSORT guidelines for clinical trials ( Moher et al. 2010) are very clear on the importance of having a single primary outcome:

*All RCTs assess response variables, or outcomes (end points), for which the groups are compared. Most trials have several outcomes, some of which are of more interest than others. The primary outcome measure is the pre-specified outcome considered to be of greatest importance to relevant stakeholders (such a patients, policy makers, clinicians, funders) and is usually the one used in the sample size calculation. Some trials may have more than one primary outcome. Having several primary outcomes, however, incurs the problems of interpretation associated with multiplicity of analyses and is not recommended.*

This advice often creates a dilemma for the researcher: in many situations there are multiple measures that could plausibly be used to index the outcome. A common solution is to apply a Bonferroni correction to the alpha level used to test significance of individual measures, but this is over-conservative if, as is usually the case, the different outcomes are intercorrelated. Alternative methods are to adopt some process of data reduction, such as extracting a principal component from the measures that can be used as the primary outcome, or using a permutation test to derive exact probability of an observed pattern of results. Here I explore a further, very simple, option which I term the “Adjust NVar” approach. The idea is that if one has a suite of outcomes, instead of adjusting the alpha level, one can adjust the number of outcomes that are required to achieve significance at the conventional alpha level of .05 to maintain an overall familywise error rate of 1 in 20 or less.

To illustrate the idea with a realistic example, suppose we are reading a report of a behavioural intervention that is designed to improve language and literacy, and there are 6 measures where we might plausibly expect to see some benefit. The researchers report that none of the outcomes achieve the Bonferroni-adjusted significance criterion of p < .008, but two of them reach significance at p < .05. Should we dismiss the trial as showing no benefit? We can use the binomial theorem to check the probability of obtaining this result if the null hypothesis is true and the measures are independent: it is 0.033, clearly below the 5% alpha level. But what if the measures are intercorrelated? That is often the case: indeed, it would be very unusual for a set of outcome measures to be independent. A thought experiment helps here. Suppose we had six measures that were intercorrelated at .95 - in effect they would all be measures of the same thing, and so if there was a real effect, most of the measures should show it. Extending this logic in a more graded way, the higher the correlation between the measures, the more measures would need to reach the original significance criterion to maintain the overall significance level below .05.

A simulation script was developed to test these intuitions and to obtain estimates of:

i) the minimum number of outcome variables in a suite that would maintain the overall familywise error rate at 1 in 20, if each individual measure was evaluated at the significance criterion of .05. This we term MinNSig.
ii) the power to detect a true effect, if the criterion for rejection the null hypothesis was based on the value of MinNSig identified at step A.

## Methods

Correlated variables were simulated using in the R programming language ( R Core Team 2020) ( R Project for Statistical Computing, RRID:SCR_001905). The script to generate and analyse simulated data is available on https://github.com/oscci/MinSigVar. Initially, two approaches to modeling correlated variables were compared, but differences between them proved to be trivial, and so only one is reported here.

### Method for simulating outcomes

The *mvrnorm* function of the *MASS* package ( Modern Applied Statistics with S, RRID:SCR_019125) was used to generate a set of 12 outcome variables with a specified covariance matrix. For simplicity, all variables were simulated as random normal deviates with SD of 1, and the covariance matrix had a prespecified correlation, r, in all off-diagonal elements. The correlation varied across runs from 0 to .8 in steps of .2, and the number of simulated cases varied from 20 to 110 in steps of 30. Outcomes for Intervention (I) and Control (C) groups differed only in terms of the mean, which was always zero for the group C, and a given effect size, e, for group I. The average observed effect size for all measures in a given condition was computed and used as the basis for comparisons of efficiency between single and multiple measure scenarios.

This method is simple but can lead to unrealistic data: in particular it is possible to have a set of outcomes that are independent of one another (r = 0) yet all having the same effect size. In real-world data, one would expect outcomes to be correlated, especially those that all showed an impact of intervention. Conversely, if a set of outcomes was very highly intercorrelated, then we would expect them all to show a similar intervention effect.

An alternative approach was evaluated to consider such cases, in which the set of 12 outcome measures are simulated as indicators of an underlying latent variable, which mediates the intervention effect. This can be achieved by first simulating a latent variable, with an effect size of either zero, for group C, or e for group I. Observed outcome measures are then simulated as having a specific correlation with the latent variable - i.e. the correlation determines the extent to which the outcomes act as indicators of the latent variable. This can be achieved using the formula:

$$r*L+\sqrt{1-r^{2}}*E$$

where r is the correlation between latent variable (L) and each outcome, and L is a vector of random normal deviates that is the same for each outcome variable, while E (error) is a vector of random normal deviates that differs for each outcome variable. Note that when outcome variables are generated this way, the mean intercorrelation between them will be r ^2^. Thus if we want a set of outcome variables with mean intercorrelation of .4, we need to specify r in the formula above as sqrt(r) = .632. Furthermore, the effect size for the simulated variables will be lower than for the latent variable: to achieve an effect size, e, for the outcome variables, it is necessary to specify the effect size for the latent variable, e _l_, as e/r ^2^. It was found that when this is done, the results with this method were closely similar to those obtained using MASS, for the range of correlations and effect sizes considered here. The exception is for the case where r = 0, which is not computuable with this method - i.e. it is not possible to have a set of outcomes that are indicators of the same latent factor but which are uncorrelated. As noted above, the case where r = 0 is unrealistic in any case, and so for the simulations reported here, the lowest value of r that was included was r = .2.

### Data reduction

The size of the suite of outcome variables entered into later analysis ranged from 2 to 12. For each suite size, principal components were computed from data from the C and I groups combined, using the base R function *prcomp* from the *stats* package ( R Core Team, 2020). Thus, PC2 is a principal component based on the first two outcome measures, PC4 based on the first four outcome measures, and so on.

Power of analyses based on the principal components was compared with power obtained using the Adjust NVar approach, as specified below.

### Simulation parameters

10,000 simulations were run for each combination of:

- sample size per group, ranging from 20 to 110 in steps of 30
- correlation between outcome variables, ranging from .2 to .8 in steps of .2
- true effect size, taking values of 0, .3, .5, or .7.

The data generated from each combination of conditions was used to derive results for different sizes of suites of outcome variables, ranging from 2 to 12. Thus, the analysis was first conducted on the first 2 outcome measures, then on the first 3 outcome measures, and so on.

For each set of conditions, on each run, a one-tailed t-test was conducted to obtain a p-value for the comparison between C and I groups, assuming C would be lower. The p-values for outcome measures were rank ordered for each run and each suite size.

### Identifying MinNSig

To obtain MinNSig, the results were filtered to include only the runs where the null hypothesis was true, i.e. effect size = 0. Then, the proportion of p-values less than .05 was calculated for each rank for each number of outcome variables, to find the highest rank at which the overall proportion was less than .05. This is the MinNSig.

Table 1 gives a toy example of the logic, using the case where we have either 2 or 4 outcome measures. Columns V1 to V4 show p-values for the t-test comparing the two groups each of the 4 outcome measures. Columns r2.1 and r2.2 show the same p-values rank ordered for just the first two measures; columns r4.1 to r4.4 show the p-values rank ordered for all 4 outcomes. We can then count the number of p-values that are below .05 for all runs (1000 in this case) for each ranked position. With 2 outcomes, if we take just the first ranked (lowest) p-value, the proportion lower than .05 is around .10. For the 2nd ranked p-value, the proportion drops below .05, to .002. Thus, we set MinNSig to 2.

**Table 1.** Demonstration of how MinNSig is determined. V1 to V4 are p-values from one-sided t-test, one row for each run of simulation in a given condition. Columns with prefix r2 or r4 show the same p-values rank ordered for either the first two columns, or the first four columns. The final two rows show the number and the proportion of values falling below .05 for that column.

run	V1	V2	V3	V4	r2.1	r2.2	r4.1	r4.2	r4.3	r4.4
1	0.877	0.569	0.642	0.661	0.569	0.877	0.569	0.642	0.661	0.877
2	0.272	0.367	0.841	0.954	0.272	0.367	0.272	0.367	0.841	0.954
3	0.993	0.116	0.249	0.414	0.116	0.993	0.116	0.249	0.414	0.993
4	0.366	0.613	0.73	0.401	0.366	0.613	0.366	0.401	0.613	0.73
5	0.735	0.559	0.329	0.715	0.559	0.735	0.329	0.559	0.715	0.735
6	0.781	0.561	0.123	0.052	0.561	0.781	0.052	0.123	0.561	0.781
7	0.805	0.779	0.989	0.991	0.779	0.805	0.779	0.805	0.989	0.991
8	0.97	0.233	0.777	0.151	0.233	0.97	0.151	0.233	0.777	0.97
9	0.129	0.432	0.215	0.005	0.129	0.432	0.005	0.129	0.215	0.432
10	0.897	0.582	0.588	0.64	0.582	0.897	0.582	0.588	0.64	0.897
...	...	...	...	...	...	...	...	...	...	...
999	0.034	0.335	0.238	0.208	0.034	0.335	0.034	0.208	0.238	0.335
1000	0.045	0.086	0.176	0.001	0.045	0.086	0.001	0.045	0.086	0.176
N < .05	.	.	.	.	100	2	185	14	0	0
p < .05	.	.	.	.	0.1	0.002	0.185	0.014	0	0

We can then turn to the case where we have four outcomes: the proportion of the 1st ranked p-values below .05 is .185; the proportion of the second ranked below .05 is .014. Thus again, we set MinNSig to 2. As noted above, when the correlation between variables is zero, we can use the binomial theorem to compute values in the final row; however, when variables are intercorrelated, more p-values will be below .05, and so MinNSig may be higher.

Because MinNSig moves in quantum steps, the effective familywise error rate is often lower than .05. For instance, in the example above with a suite of four outcome measures, MinNSig is set to 2, but this gives p = .014, rather than .05.

### Computing power using Adjust NVar

For each run of the simulation, and each number of outcome measures, we take the value of MinNSig from the previous step and compute the proportion of p-values below .05, depending on the effect size, sample size and correlation between measures. For effect sizes above zero, this proportion corresponds to the statistical power.

Power using Adjust NVar can be compared to:

- power obtained with a single outcome measure for the same effect size and sample size
- power obtained by using the principal component extracted for this set of outcome measures

## Results

### MinNSig

Table 2 shows results from a simulation of the Adjust NVar approach, with the values in the body of the table showing MinNSig, the minimum number of measures that would maintain the overall familywise error rate at 1 in 20, if each individual measure was evaluated at the significance criterion of .05. Because the t-test statistic used to determine p-values is adjusted for sample size, these values are independent of numbers of subjects. In principle, researchers could use Table 2 to specify in their research protocol the minimum number of outcomes that would reach their significance level in order for the null hypothesis to be rejected.

**Table 2.** Values of MinNSig for different suite sizes of outcomes. Entries in body of table show smallest N variables reaching p < .05 that preserve familywise error rate at .05 or less. N prefix denotes suite size for a set of outcomes. Corr indicates correlation between outcomes.

corr	N2	N3	N4	N5	N6	N7	N8	N9	N10	N11	N12
0.0	2	2	2	2	2	2	3	3	3	3	3
0.2	2	2	2	2	3	3	3	3	3	3	4
0.4	2	2	2	3	3	3	3	3	4	4	4
0.6	2	2	2	3	3	3	4	4	4	5	5
0.8	2	2	3	3	3	4	4	4	5	5	6

### Power of Adjust NVar approach

**Figure 1.**
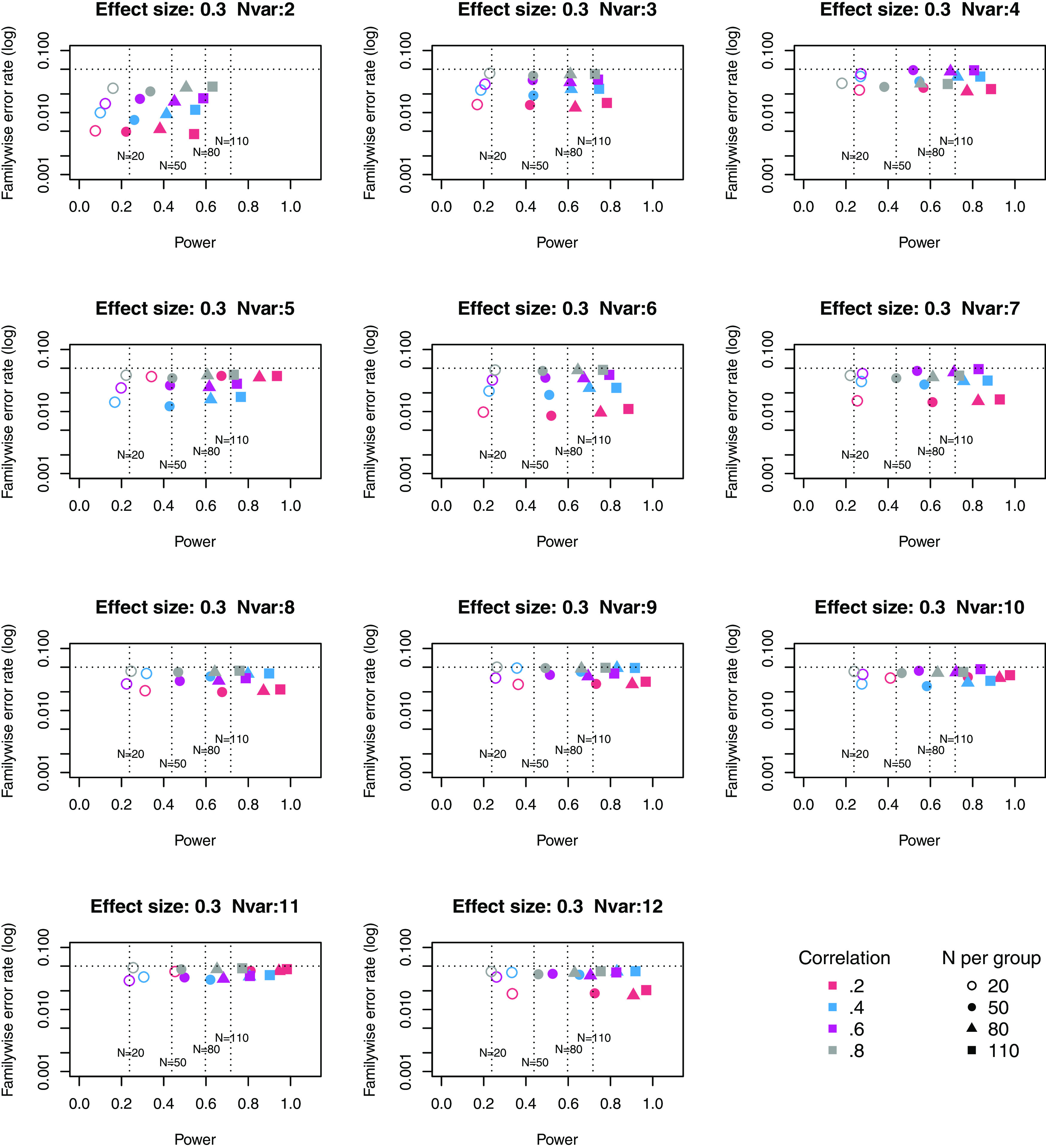
Power × Familywise error rate using Adjust NVar method, for small effect size. Symbols denote sample size per group, and colours denote correlation between outcomes (see Key). Vertical dotted lines show power for single outcome at different sample sizes. Horizontal line shows type I error rate of .05.

**Figure 2.**
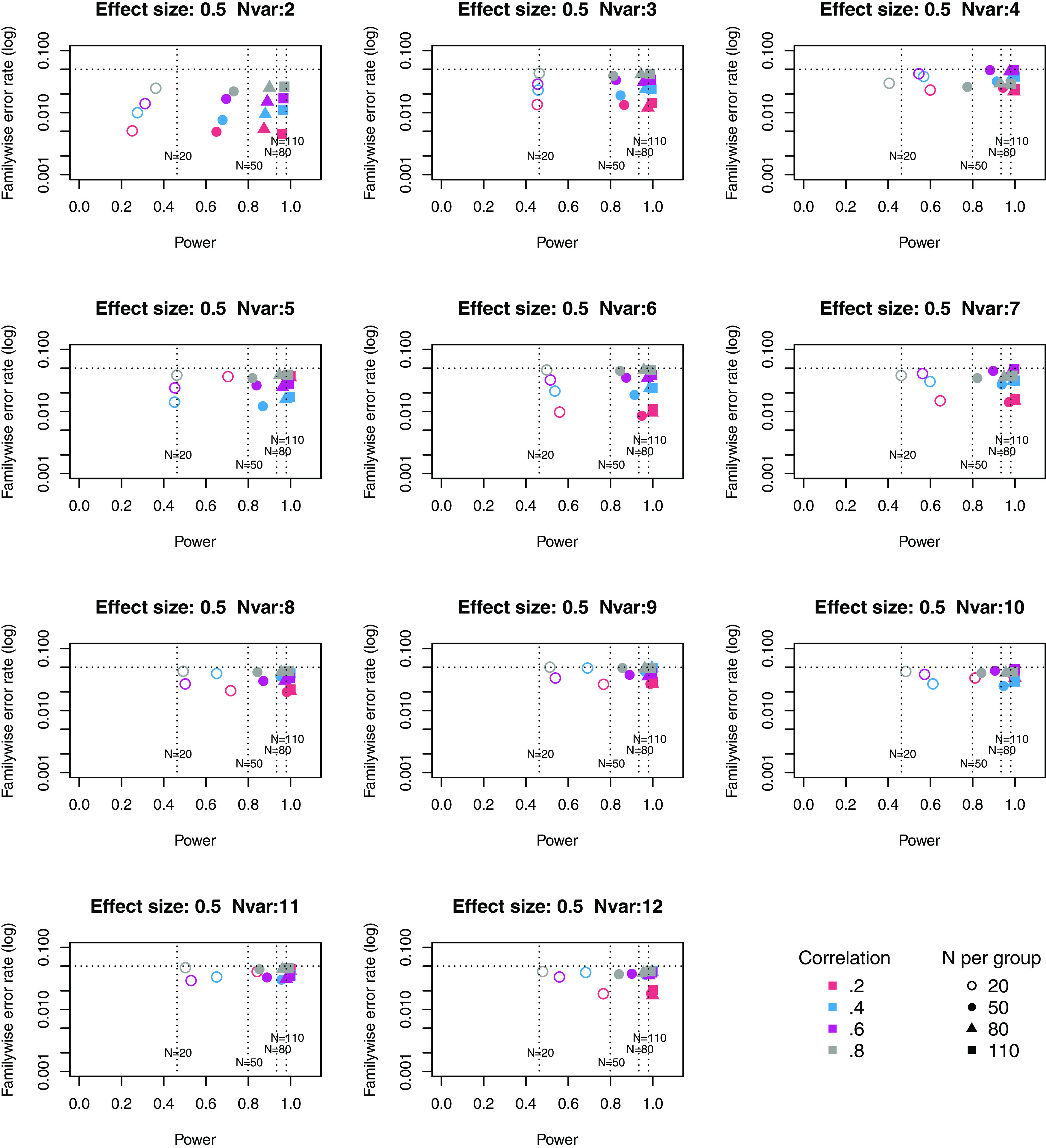
Power × Familywise error rate using Adjust NVar method, for medium effect size. Symbols denote sample size per group, and colours denote correlation between outcomes (see Key). Vertical dotted lines show power for single outcome at different sample sizes. Horizontal line shows type I error rate of .05.

**Figure 3.**
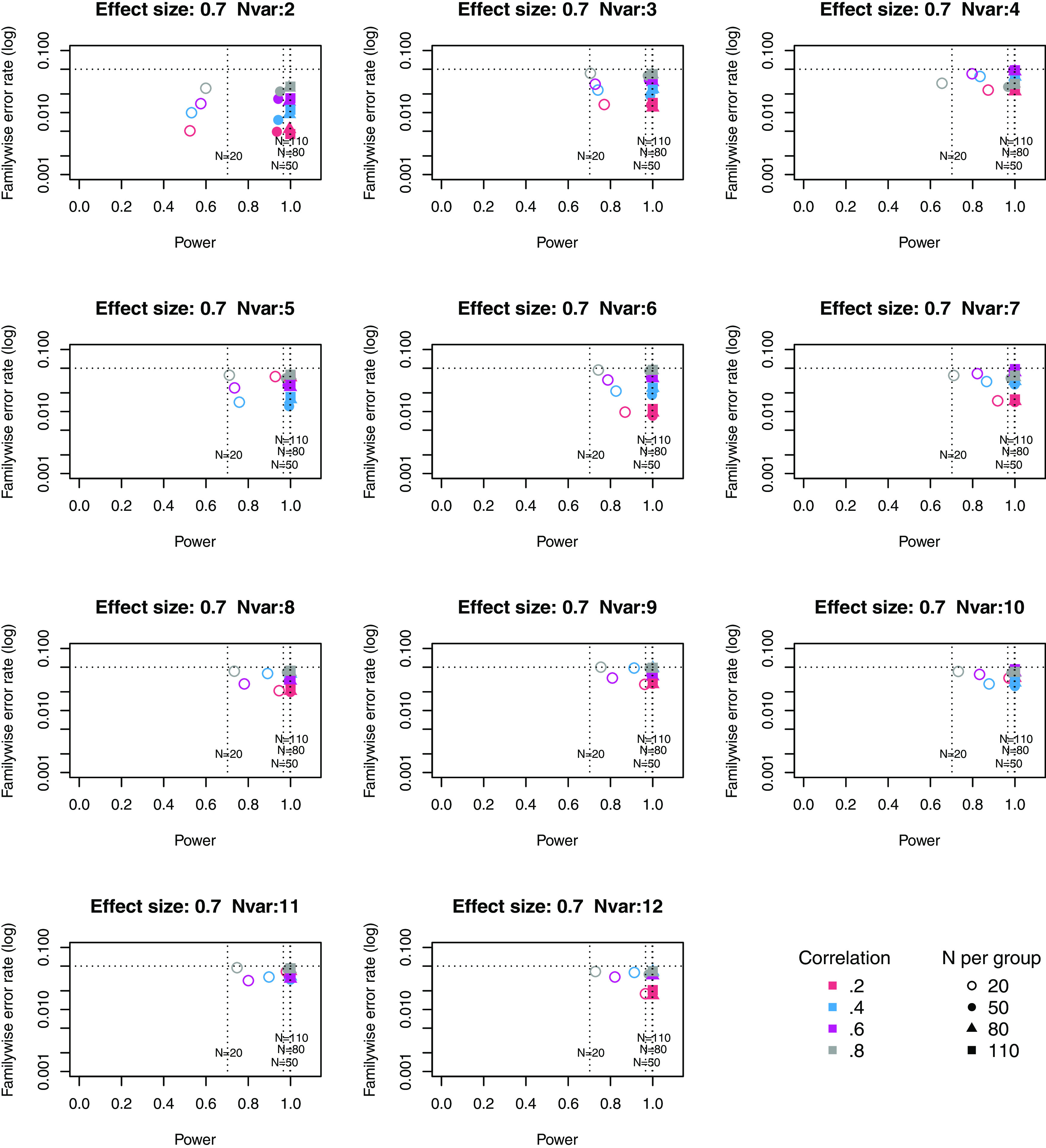
Power × Familywise error rate using Adjust NVar method, for large effect size. Symbols denote sample size per group, and colours denote correlation between outcomes (see Key). Vertical dotted lines show power for single outcome at different sample sizes. Horizontal line shows type I error rate of .05.

Full tables of results for all combinations of parameters are provided in Extended Data. Figures 1 to 3 plot power vs familywise error rate for different sizes of suite of outcome measures.

For these plots, we see power for small, medium and large effect sizes (corresponding to Cohen’s d of .3, .5 and .7). An efficient method is one that gives power of .8 or above, and a familywise error rate of .05 or less, i.e. the results should cluster in the bottom right quadrant. Power, which depends on sample size, is shown for a study with a single outcome in the vertical dotted lines, with an alpha of .05 shown in the horizontal dotted line. We can compare by eye how well Adjust Nmin with multiple outcomes compares with a single outcome for the same sample size. With just two outcome measures we obtain a very low familywise error rate but power is generally worse than for the single outcome case, except when the effect size is large. This is because when using Adjust NMin with two outcomes, both outcomes have to achieve p < .05. With three outcomes, again Adjust NMin requires we have at least two individual outcomes with p < .05: this gives power equivalent to that of a single variable, but a lower familywise error rate is achieved. When the number of outcomes is four or more, the benefit of Adjust NMin over a single outcome becomes more evident, with higher power coupled with a lower familywise error rate. The specific results depend also on the intercorrelation between outcomes (which in turn influences the MinNSig value, see Table 2): a moderate level of intercorrelation (between .4 and .6) generally gives an efficient measure.

**Figure 4.**
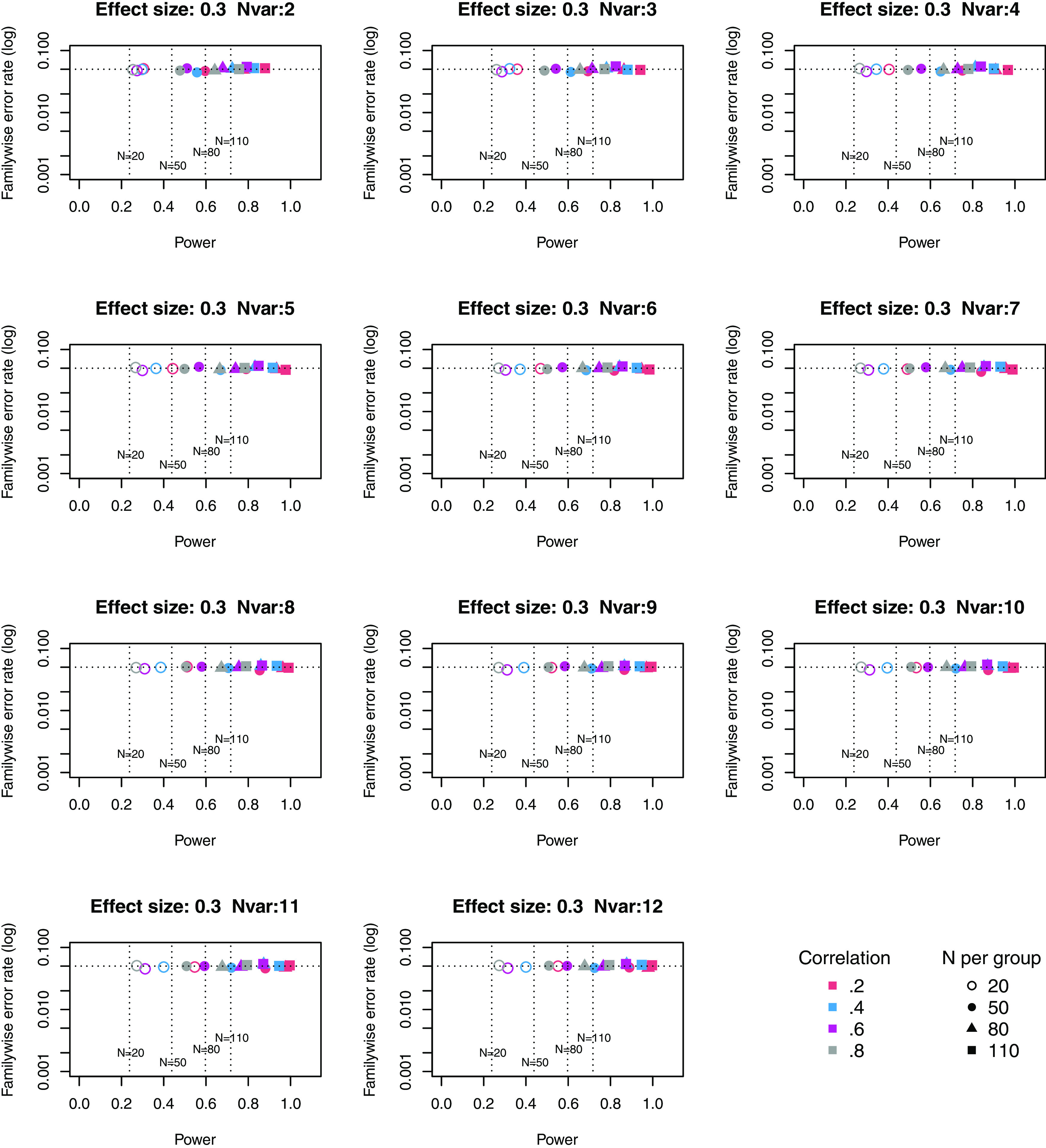
Power × Familywise error rate using 1st principal component, for small effect size. Symbols denote sample size per group, and colours denote correlation between outcomes (see Key). Vertical dotted lines show power for single outcome at different sample sizes. Horizontal line shows type I error rate of .05.

**Figure 5.**
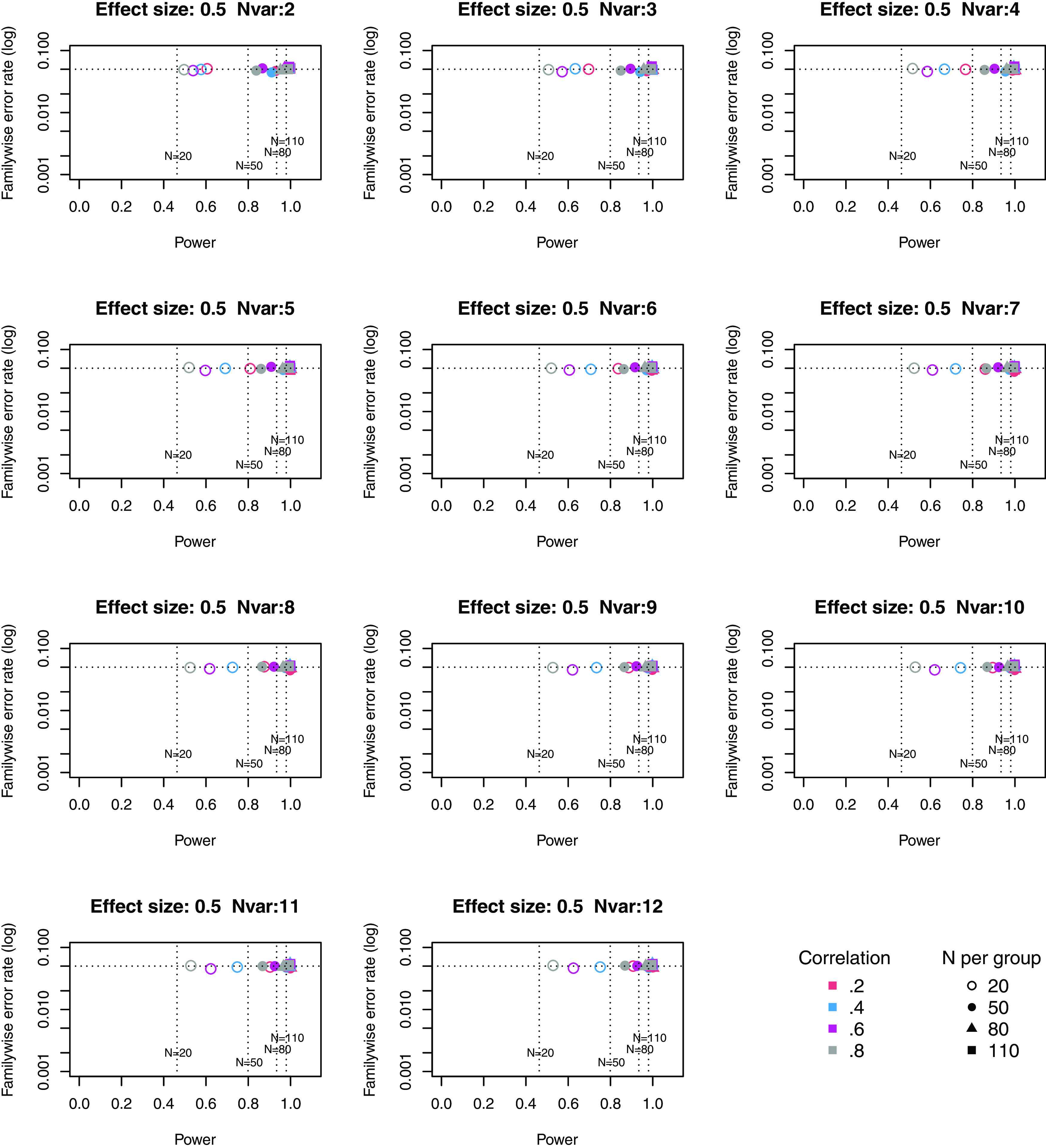
Power × Familywise error rate using 1st principal component, for medium effect size. Symbols denote sample size per group, and colours denote correlation between outcomes (see Key). Vertical dotted lines show power for single outcome at different sample sizes. Horizontal line shows type I error rate of .05.

**Figure 6.**
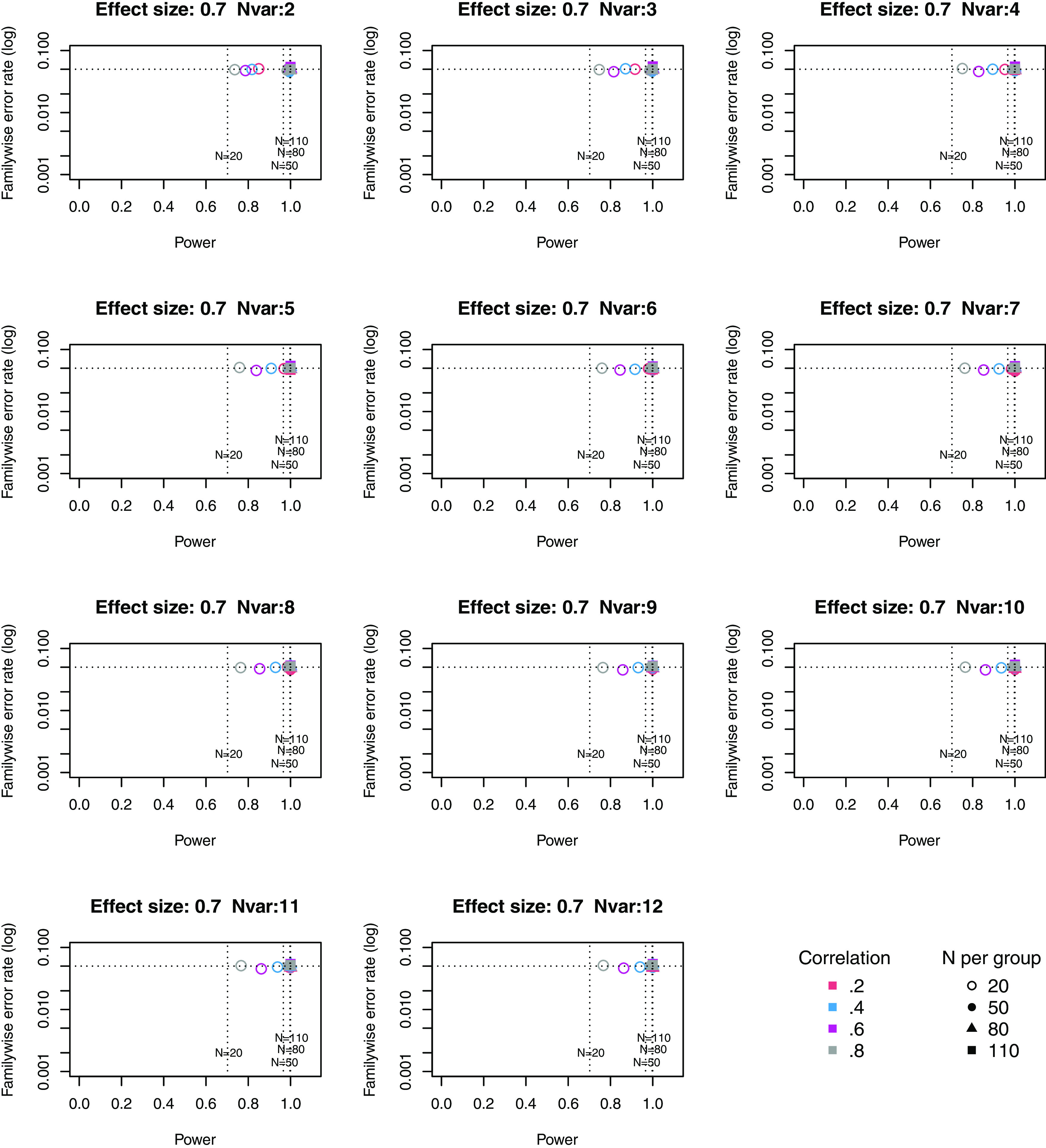
Power × Familywise error rate using 1st principal component, for large effect size. Symbols denote sample size per group, and colours denote correlation between outcomes (see Key). Vertical dotted lines show power for single outcome at different sample sizes. Horizontal line shows type I error rate of .05.

Figures 4 to 6 give equivalent plots for power from principal components.

The Principal Components plots show all points are to the right of the vertical line denoting power from a single outcome, i.e. this method achieves higher power than a single outcome measure for each size of suite of outcomes. Familywise error rate clusters around .05. If we compare how Adjust NVar compares with Principal Components, with three or more outcomes the power is generally slightly lower, but the tradeoff between power and familywise error (expressed as a ratio) is higher for Adjust NVar.

## Discussion

The logic of conventional multiple testing is turned on its head with the Adjust NVar approach, in that instead of adjusting the p-value used for significance (as in the Bonferroni correction, or methods based on False Discovery Rate), we adjust the number of individual outcome measures that we need to reach the intended significance criterion. This value can be easily computed using the binomial theorem for a given suite size of outcomes if the measures are uncorrelated, but in the context of intervention trials uncorrelated measures is an unrealistic assumption.

One advantage of this approach is that it is more compatible with trials of interventions that are expected to affect a range of related processes, as is common in some fields such as education or speech and language therapy. In such cases, the need to specify a single primary outcome tends to create difficulties, because it is often unclear which of a suite of outcomes is likely to show an effect. Note that the Adjust NVar approach does not give the researcher free rein to engage in p-hacking: the larger the suite of measures included in the study, the higher the value of MinNSig will be. It does, however, remove the need to put all one’s eggs in one basket by pre-specifying one measure as the primary outcome.

A second advantage is that in effect, by including multiple outcome measures, one can improve the efficiency of a study, in terms of the trade-off between power and familywise errors. A set of outcome measures may be regarded as imperfect proxy indicators of an underlying latent construct, so we are in effect building in a degree of within-study replication if we require that more than one measure shows the same effect in the same direction before we reject the null hypothesis.

The comparison with power and familywise error rate from principal components shows that the latter approach is more consistent in improving power over a study with a single outcome than the Adjust NVar approach, regardless of the size of the suite of outcomes, but it does not influence familywise error rate. Variation in familywise error rate is a consequence of the quantum nature of the adjustment with Adjust NVar, where the same value of MinNVar may be used with varying sizes of outcome suite, which can lead to values of familywise error rate well below .05. For instance, obtaining two p-values below .05 in a suite of two outcomes is a more unusual circumstance than obtaining two values this extreme in a suite of three or four outcomes. Nevertheless, the ratio of power to familywise error is generally higher for Adjust NVar than for principal components.

A possible disadvantage of Adjust NVar over principal components is that this approach is likely to tempt researchers to interpret specific outcomes that fall below the .05 threshold as meaningful. They may be, of course, but this simulation demonstrates that when we create a suite of outcomes that differ only by chance, it is common for only a subset of them to reach the significance criterion. Any recommendation to use Adjust NVar should be accompanied by a warning that a suite of outcomes should be selected as representative of the underlying construct the intervention is designed to influence, in effect serving as replicate measures, all of which should be equally promising as indicators of an intervention effect. If a subset of outcomes show an effect of intervention, this could be due to chance. It would be necessary to run a replication to have confidence in a particular pattern of results.

It is also worth noting that results obtained with this approach depend crucially on assumptions embodied in the simulation that is used to derive predictions. Outcome measures simulated here are normally distributed, and uniform in their covariance structure. It would be possible to generate datasets with different underlying covariance structures to be tested in the same way, but that is beyond the scope of this paper.

Perhaps the main advantage of this approach is that once the values of MinNSig have been specified (as in Table 2), the method is very simple to apply, and could be used in two ways. First, it can be used *a priori* to specify in a protocol the number of outcomes that would need to achieve the conventional .05 level of significance, in order for the intervention to be deemed effective. This assumes that the researcher already has a rough idea of the degree of intercorrelation between outcome measures, but a range somewhere between .4 and .6 is a reasonable assumption for many behavioural studies. Pre-registering a specific level of MinSigN would help guard against a tendency to explore different kinds of correction for multiple hypothesis testing only after viewing the data ( Lazic 2021).

Second, the simplicity of the approach makes it useful for evaluating published studies that report multiple outcomes but may not have been analysed optimally. We started with the example of a study where there were six outcome measures, none of which met the Bonferroni-corrected significance level of .05/6 = .008, but two of which met p < .05. From Table 2 we can see that to be confident in a true intervention effect, assuming correlated outcomes, then at least three out of six outcomes need to be significant at the .05 level. In this case, therefore, we do not reject the null hypothesis.

In sum, the Adjust NVar method shows how inclusion of multiple outcomes can be a positive strategy in intervention studies and can give stronger statistical evidence than a single outcome, provided that attention is paid to the need for several outcomes to reach a significance threshold.

## Data availability statement

### Extended data

OSF: Adjust NVar. https://doi.org/10.17605/OSF.IO/5T4SE. ( Bishop, 2021).

This project contains the following extended data.

• Extended data.docx (Word version of powertab_methodM.csv, showing power for each combination of parameters, with separate subtables for individual variables ( Figures 1-3) and principal components ( Figures 4- 6).

Data are available under the terms of the Creative Commons Zero “No rights reserved” data waiver (CC0 1.0 Public domain dedication).

## Software availability

The script to generate and analyse simulated data is available on https://github.com/oscci/MinSigVar.

The OSF project contains the following files.

• toybit.csv - corresponds to Table 1
• MinNSig_methodM.csv - corresponds to Table 2
• p_1sided_methodM_allN_allES_allcorr_maxn12_nsim10000_nstep1.csv - output of simulation of 10000 runs of Multiple_outcomes.Rmd script
• Extended data.docx - word version of powertab_methodM.csv
• powertab_methodM.csv - csv version of Extended Data table, with individual variables in columns N1-N12, and principal components in columns PC2-PC12

## References

[ref1] Bishop DVM : Adjust NVar. 2021, September 24. Reference Source

[ref2] Lazic SE : Why Multiple Hypothesis Test Corrections Provide Poor Control of False Positives in the Real World. *arXiv:2108.04752 [q-Bio, Stat]* .2021; (August). Reference Source 10.1037/met0000678 39570698

[ref3] Moher D Hopewell S Schulz KF : CONSORT 2010 Explanation and Elaboration: Updated Guidelines for Reporting Parallel Group Randomised Trials. *BMJ (Clinical Research Ed.)* .2010;340(March):c869. 10.1136/bmj.c869 PMC2844943 20332511

[ref4] R Core Team: *R: A Language and Environment for Statistical Computing* . Vienna, Austria: R Foundation for Statistical Computing;2020. Reference Source

[ref5] Venables WN Ripley BD : *Modern Applied Statistics with S* . Fourthed. New York: Springer;2002. 0-387-95457-0.

