# Using multiple outcomes in intervention studies: improving power while controlling type I errors

**DOI:** 10.12688/f1000research.73520.3

**Published:** 2023-11-06

**Authors:** Dorothy V. M. Bishop

**Affiliations:** 1 Department of Experimental Psychology, University of Oxford, Oxford, Oxon, OX2 6GG, UK

**Keywords:** intervention, methodology, statistics, correlated outcomes, power, familywise error rate, multiple comparisons

## Abstract

Background

The CONSORT guidelines for clinical trials recommend use of a single primary outcome, to guard against the raised risk of false positive findings when multiple measures are considered. It is, however, possible to include a suite of multiple outcomes in an intervention study, while controlling the familywise error rate, if the criterion for rejecting the null hypothesis specifies that N or more of the outcomes reach an agreed level of statistical significance, where N depends on the total number of outcome measures included in the study, and the correlation between them.

Methods

Simulations were run, using a conventional null-hypothesis significance testing approach with alpha set at .05, to explore the case when between 2 and 12 outcome measures are included to compare two groups, with average correlation between measures ranging from zero to .8, and true effect size ranging from 0 to .7. In step 1, a table is created giving the minimum N significant outcomes (MinNSig) that is required for a given set of outcome measures to control the familywise error rate at 5%. In step 2, data are simulated using MinNSig values for each set of correlated outcomes and the resulting proportion of significant results is computed for different sample sizes,correlations, and effect sizes.

Results

The Adjust NVar approach can achieve a more efficient trade-off between power and type I error rate than use of a single outcome when there are three or more moderately intercorrelated outcome variables.

Conclusions

Where it is feasible to have a suite of moderately correlated outcome measures, then this might be a more efficient approach than reliance on a single primary outcome measure in an intervention study. In effect, it builds in an internal replication to the study. This approach can also be used to evaluate published intervention studies.

## Issues raised by inclusion of multiple outcomes

The CONSORT guidelines for clinical trials ( Moher et al., 2010) are very clear on the importance of having a single primary outcome:

*All RCTs assess response variables, or outcomes (end points), for which the groups are compared. Most trials have several outcomes, some of which are of more interest than others. The primary outcome measure is the pre-specified outcome considered to be of greatest importance to relevant stakeholders (such as patients, policy makers, clinicians, funders) and is usually the one used in the sample size calculation. Some trials may have more than one primary outcome. Having several primary outcomes, however, incurs the problems of interpretation associated with multiplicity of analyses and is not recommended.*

This advice often creates a dilemma for the researcher: in many situations there are multiple measures that could plausibly be used to index the outcome ( Vickerstaff, Ambler, King, Nazareth, & Omar, 2015). If we have several outcomes and we would be interested in improvement on any measure, then we need to consider the familywise error rate, i.e. the probability of at least one false positive in the whole set of outcomes. For instance, if we want to set the false positive rate, alpha to .05, and we have six independent outcomes, none of which is influenced by the intervention, the probability that none of the tests of outcome effects is significant will be .95^6, which is .735. Thus the probability that at least one outcome is significant, the familywise error rate, is 1-.735, which is .265. In other words, in about one quarter of studies, we would see a false positive when there is no true effect. The larger the number of outcomes, the higher the false positive rate.

A common solution is to apply a Bonferroni correction by dividing the alpha level by the number of outcome measures - in this example .05/6 = .008. This way the familywise error rate is kept at .05. But this is over-conservative if, as is usually the case, the various outcomes are intercorrelated.

Various methods have been developed to address the problem of multiple testing. One approach is to adopt some process of data reduction, such as extracting a principal component from the measures that can be used as the primary outcome. Alternatively, a permutation test can be used to derive exact probability of an observed pattern of results. Neither approach, however, is helpful if the researcher is evaluating a published paper where an appropriate correction has not been made. These could be cases where no correction is made for multiple testing, risking a high rate of false positives, or where Bonferroni correction has been applied despite using correlated outcomes, which will be overconservative in rejecting the null hypothesis. The goal of the current article is to provide some guidance for interpretation of published papers where the raw data are not available for recomputation of statistics.

Vickerstaff et al. (2015) reviewed 209 trials in neurology and psychiatry, and found that 60 reported multiple primary outcomes, of which 45 did not adjust for multiplicity. Those that did adjust mostly used the Bonferroni correction. Thus it would appear that many researchers feel the need to include several outcomes, but this is not always adjusted for appropriately. The goal of the current article is to provide some guidance for interpretation of published papers where the raw data are not available for recomputation of statistics.

In a review of an earlier version of this paper, Sainani (2021) pointed out that the MEff statistic, originally developed in the field of genetics by Cheverud (2001) and Nyholt (2004), provided a simple way of handling this situation. With this method, one computes eigenvalues from the correlation matrix of outcomes, which reflect the degree of intercorrelation between them. The mathematical definition of an eigenvalue can be daunting, but an intuitive sense of how it relates to correlations can be obtained by considering the cases shown in Table 1. This shows how eigenvalues vary with the correlation structure of a matrix, using an example of six outcome measures. The number of eigenvalues, and the sum of the eigenvalues, is identical to the number of measures. Let us start by assuming a matrix in which all off-diagonal values are equal to *r.* It can be seen that when the correlation is zero, each eigenvalue is equal to one, and the variance of the eigenvalues is zero. When the correlation is one, the first eigenvalue is equal to six, all other eigenvalues are zero, and the variance of the eigenvalues is six. As correlations increase from .2 to .8, the size of the first eigenvalue increases, and that of the other eigenvalues decreases.

**Table 1.** Eigenvalues, MEff and AlphaMEff with 6 outcome variables.

r	Eigen1	Eigen2	Eigen3	Eigen4	Eigen5	Eigen6	Var	MEff	AlphaMEff
0	1.0	1.0	1.0	1.0	1.0	1.0	0.00	6.0	0.008
0.2	2.0	0.8	0.8	0.8	0.8	0.8	0.24	5.8	0.009
0.4	3.0	0.6	0.6	0.6	0.6	0.6	0.96	5.2	0.010
0.6	4.0	0.4	0.4	0.4	0.4	0.4	2.16	4.2	0.012
0.8	5.0	0.2	0.2	0.2	0.2	0.2	3.84	2.8	0.018
1	6.0	0.0	0.0	0.0	0.0	0.0	6.00	1.0	0.050

In Table 1, *r* is the intercorrelation between the six outcomes, Eigen1 - Eigen6, are the eigenvalues, and Var is the variance of the six Eigenvalues, which is used to compute MEff (the effective number of comparisons) from the formula:

MEff=1+(N-1)*(1-(Var(Eigen)/N)

where N is the number of outcome measures, and Eigen is the set of N eigenvalues.

This value is then used to compute the corrected alpha level, AlphaMEff. Assuming we set alpha to .05, AlphaMEff is .05 divided by MEff. One can see that this value is equivalent to the Bonferroni-corrected alpha (.05/6) when there is no correlation between variables, and equivalent to .05 when all variables are perfectly correlated.

Derringer (2018) provided a useful tutorial on MEff, noting that it is not well-known outside the field of genetics, but is well-suited to the field of psychology. Her preprint includes links to R scripts for computing MEff and illustrates their use in three datasets.

These resources will be sufficient for many readers interested in using MEff, but researchers may find it useful to have a look-up table for the case when they are evaluating existing studies. The goal of this paper is two-fold:

A. To consider how inclusion of multiple outcome measures affects statistical power, relative to the case of a single outcome, when appropriate correction of the familywise error rate is made using MEff. Results from MEff are compared with use of Bonferroni correction and analysis of the first component derived from Principal Components Analysis (PCA).
B. To provide a look-up table to help evaluate studies with multiple outcome measures, without requiring the reader to perform complex statistical analyses.

These goals are achieved in three sections below:

1. Power to detect a true effect using MEff is calculated from simulated data for a range of values of sample size (N), effect size (E) and the matrix of intercorrelation between outcomes (R)
2. A lookup table is provided that gives values of MEff, and associated adjusted alpha-levels for different set sizes of outcome measures, with mean pairwise correlation varying from 0 to 1 in steps of .1.
3. Use of the lookup table is shown for real-world examples of application of MEff using published articles.

### Alternative approach, MinNVar

In the original version of this manuscript ( Bishop, 2021), an alternative approach, MinNVar, was proposed, in which the focus was on the *number* of outcome variables achieving a conventional .05 level of significance. As noted by reviewers, this has the drawback that it could not reflect continuous change in probability levels, because it was based on integer values (i.e. number of outcomes). This made it overconservative in some cases, where adopting the MinNVar approach gave a familywise error rate well below .05. One reason for proposing MinNVar was to provide a very easy approach to evaluating studies that had multiple outcomes, using a lookup table to check the number of outcomes needed, depending on overall correlation between measures. However, it is equally feasible to provide lookup tables for MEff, which is preferable on other grounds, and so MinNVar is not presented here; interested readers can access the first version of this paper to evaluate that approach.

### Use of one-tailed p-values

In the simulations described here, one-tailed tests are used. Two-tailed p-values are far more common in the literature, perhaps because one-tailed tests are often abused by researchers, who may switch from a two-tailed to a one-tailed p-value in order to nudge results into significance.

This is unfortunate because, as argued by Lakens (2016), provided one has a directional hypothesis, a one-tailed test is more efficient than a two-tailed test. It is a reasonable assumption that in intervention research, which is the focus of the current paper, the hypothesis is that an outcome measure will show improvement. Of course, interventions can cause harms, but, unless those are the focus of study, we have a directional prediction for improvement.

## Methods

Correlated variables were simulated using the R programming language ( R Core Team, 2020) ( R Project for Statistical Computing, RRID:SCR_001905). The script to generate and analyse simulated data is available on https://osf.io/hsaky/. For each model specified below, 2000 simulations were run. Note that to keep analysis simple, a single value was simulated for each case, rather than attempting to model pre- vs post-intervention change. Data for the two groups were generated by the same process, except that a given effect size was added to scores of the intervention group, I, but not to the control group, C. Scores of the two groups were compared using a one-tailed t-test for each run.

Power was computed for different levels of effect size (E), correlation between outcomes ( **R**) and sample size per group (N) for the following methods:

a) Bonferroni-corrected data: Proportion of runs where p was less than the Bonferroni-corrected value for at least one outcome.
b) MEff-corrected data: Proportion of runs where p was less than AlphaMeff value for at least one outcome.
c) Principal component analysis (PCA): Proportion of runs where p was below .05 when groups I and C were compared on scores on the first principal component of PCA.

## Method for simulating outcomes

Simulating multivariate data forces one to consider how to conceptualise the relationship between an intervention and multiple outcomes. Implicit in the choice of method is an underlying causal model that includes mechanisms that lead measures to be correlated.

In the simulation, outcomes were modelled as indicators of one or more underlying latent factors, which mediate the intervention effect. This can be achieved by first simulating a latent factor, with an effect size of either zero, for group C, or E for group I. Observed outcome measures are then simulated as having a specific correlation with the latent variable - i.e. the correlation determines the extent to which the outcomes act as indicators of the latent variable. This can be achieved using the formula:

$$r*L+\sqrt{1-r^{2}}*e$$

where *r* is the correlation between latent variable ( *L*) and each outcome, and L is a vector of random normal deviates that is the same for each outcome variable, while *e* (error) is a vector of random normal deviates that differs for each outcome variable. Note that when outcome variables are generated this way, the mean intercorrelation between them will be *r* ^2^. Thus if we want a set of outcome variables with mean intercorrelation of .4, we need to specify r in the formula above as sqrt( *r*) = .632. Furthermore, the effect size for the simulated variables will be lower than for the latent variable: to achieve an effect size, E, for the outcome variables, it is necessary to specify the effect size for the latent variable, E _l_, as E/sqrt( *r*).

Note that the case where *r* = 0 is not computable with this method - i.e. it is not possible to have a set of outcomes that are indicators of the same latent factor but which are uncorrelated. The lowest value of *r* that was included was *r* = .2.

**Figure 1.**
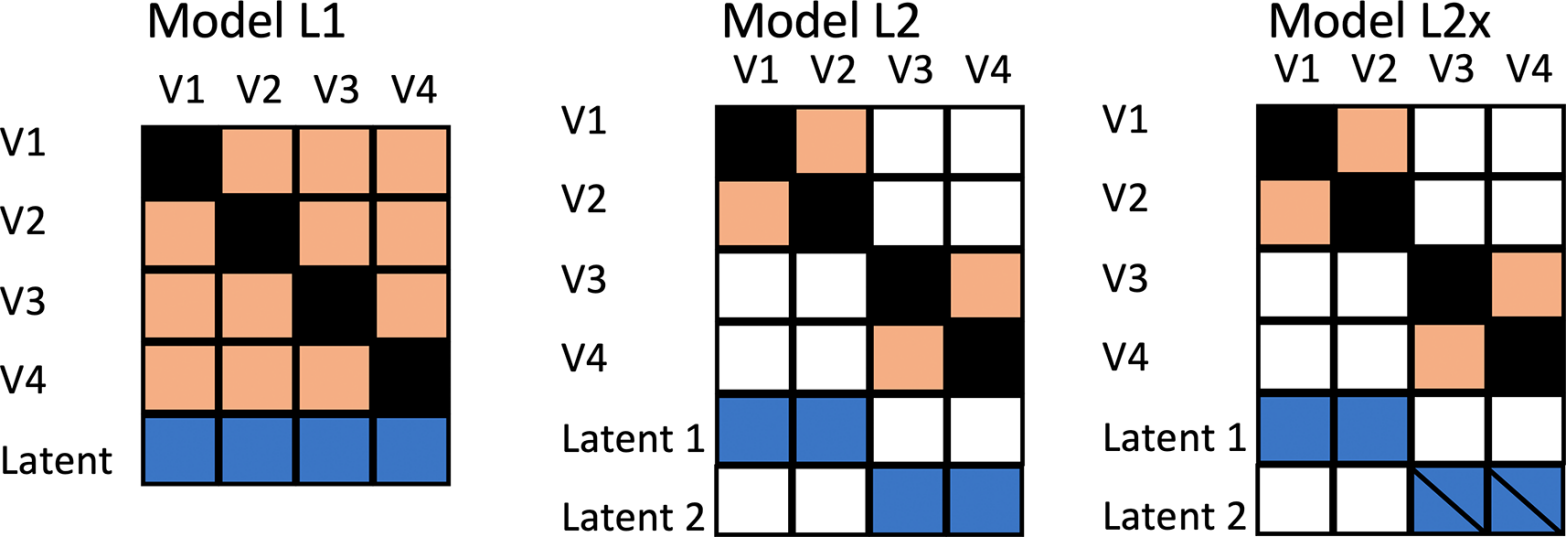
Models for data generation. Heatmap depicts correlations between observed variables V1 to V4 and Latent factors, where colour denotes association. A diagonal line through a latent factor indicates it is not related to intervention.

The initial simulation, designated as Model L1, treated all outcome measures as equivalent. In practice, of course, we will observe different effect sizes for different outcomes, but in Model L1, this is purely down to the play of chance: all outcomes are indicators of the same underlying factor, as shown in the heatmap in Figure 1, Model L1.

In two additional models, rather than being indicators of the same uniform latent variable, the outcomes correspond to different latent factors. This would correspond to the kind of study described by Vickerstaff et al. (2021), where an intervention for obesity included outcomes relating to weight and blood glucose levels. Following suggestions by Sainani (2021), a set of simulations was generated to consider relative power of different methods when there are two underlying latent factors that generate the outcomes. In Model L2, there are two independent latent factors, both affected by intervention. In Model L2×, the intervention only influences the first latent factor. The computational approach was the same as for Model L1, but with two latent factors, each used to generate a block of variables. The two latent factors are uncorrelated.

The size of the suite of outcome variables entered into later analysis ranged from 2 to 8. For each suite size, principal components were computed from data from the C and I groups combined, using the base R function *prcomp* from the *stats* package ( R Core Team, 2020). Thus, PC2 is a principal component based on the first two outcome measures, PC4 based on the first four outcome measures, and so on.

## Results

### Power calculations

**Figure 2.**
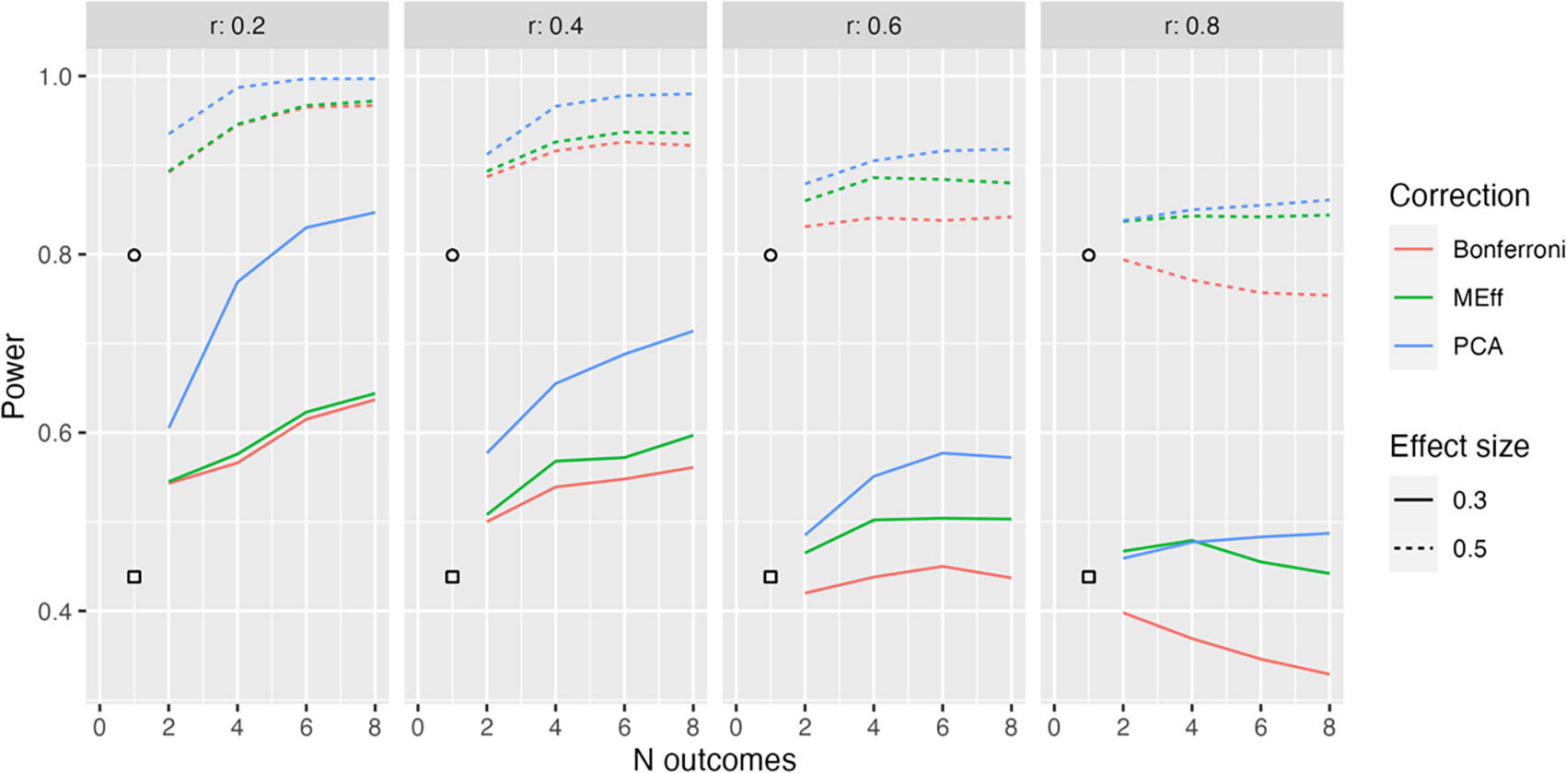
Model L1, 50 per group. Power in relation to number of outcome measures (N outcomes), intercorrelation between outcomes (column headers), type of Correction, and Effect size. The square, circle and triangle symbols represent the power for a single outcome measure with effect size .3 and .5 respectively.

**Figure 3.**
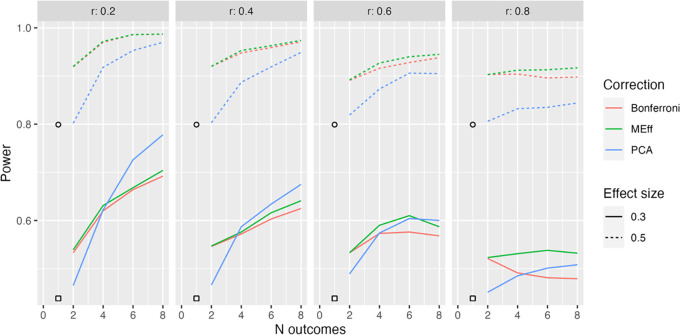
Model L2: 50 per group. Power in relation to number of outcome measures (N outcomes), intercorrelation between outcomes (column headers), type of Correction, and Effect size. The square, circle and triangle symbols represent the power for a single outcome measure with effect size .3 and .5 respectively.

**Figure 4.**
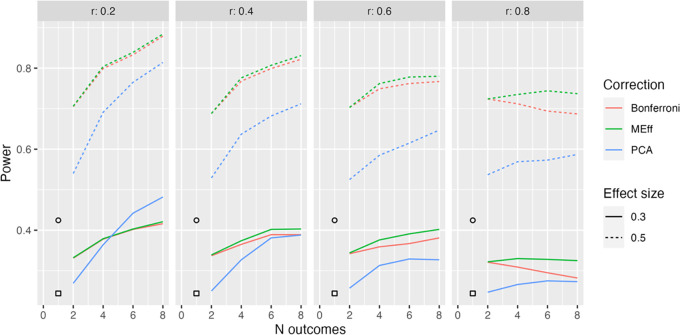
Model L2x: 50 per group. Power in relation to number of outcome measures (N outcomes), intercorrelation between outcomes (column headers), type of Correction, and Effect size. The square, circle and triangle symbols represent the power for a single outcome measure with effect size .3 and .5 respectively.

Sample plots comparing power for Bonferroni correction, MEff and PCA are shown for sample size of 50 per group in Figures 2 to 4. Plots for smaller (N = 20) and larger (N = 80) sample sizes are available online ( https://osf.io/k6xyc/), and show the same basic pattern.

Figure 2 shows the simplest situation when there are between 2 and 8 outcome measures, all of which are derived from the same latent variable (Model L1). Different levels of intercorrelation between the outcomes (ranging from .2 to .8 in steps of .2) are shown in columns.

Several points emerge from inspection of this figure; first, when intercorrelation between measures is low to medium (.2 to .6), power increases as the number of outcome measures increases. Furthermore, the power is greater when PCA is used than when MEff or Bonferroni correction is applied. MEff is generally somewhat better-powered than Bonferroni, and Bonferroni has lower power than a single outcome measure when there is a large number of highly intercorrelated outcome measures ( *r* = .8).

In practice, it may be the case that outcome measures are not all reflective of a common latent factor. Figure 3 shows results from Model L2, where outcome measures form two clusters, each associated with a different latent factor (see Figure 1). Here both latent factors are associated with improved outcomes in the intervention group.

Once again, power increases with number of outcomes when there are low to modest intercorrelations between outcomes. For this method, PCA no longer has such a clear advantage. This makes sense, given that PCA will not derive a single main factor, when the underlying data structure contains two independent factors.

Figure 4 shows equivalent results for Model L2x, where we have a mixture of two types of outcome, one of which is influenced by intervention, and the other is not. This complicates calculation of power for a single variable, since, power will depend on whether we select one of the outcomes that is influenced by intervention or not. The symbols in Figure 4 show average power, assuming we might select either type of outcome with equal frequency. We see that in this situation, MEff is clearly superior to PCA except when we have a large number of outcomes, a small effect size and weak intercorrelation between outcomes.

### Deriving a lookup table

Table 2 shows corrected alpha values based on MEff, varying according to the correlation between outcome measures, and the number of outcome measures in the study. In practice, the problem for the researcher is to estimate the intercorrelation between outcome measures if this is not known.

**Table 2.** AlphaMEff for different correlation values (corr) with 2-12 outcome variables (N2 to N12), based on Model L1.

corr	N2	N3	N4	N5	N6	N7	N8	N9	N10	N11	N12
0.0	0.025	0.017	0.013	0.010	0.008	0.007	0.006	0.006	0.005	0.005	0.004
0.1	0.025	0.017	0.013	0.010	0.008	0.007	0.006	0.006	0.005	0.005	0.004
0.2	0.026	0.017	0.013	0.010	0.009	0.007	0.006	0.006	0.005	0.005	0.004
0.3	0.026	0.018	0.013	0.011	0.009	0.008	0.007	0.006	0.005	0.005	0.005
0.4	0.027	0.019	0.014	0.011	0.010	0.008	0.007	0.006	0.006	0.005	0.005
0.5	0.029	0.020	0.015	0.013	0.011	0.009	0.008	0.007	0.006	0.006	0.005
0.6	0.030	0.022	0.017	0.014	0.012	0.010	0.009	0.008	0.007	0.007	0.006
0.7	0.033	0.025	0.020	0.016	0.014	0.012	0.011	0.010	0.009	0.008	0.008
0.8	0.037	0.029	0.024	0.020	0.018	0.016	0.014	0.013	0.012	0.011	0.010
0.9	0.042	0.036	0.032	0.028	0.026	0.023	0.021	0.020	0.018	0.017	0.016
1.0	0.050	0.050	0.050	0.050	0.050	0.050	0.050	0.050	0.050	0.050	0.050

Model L1, used to generate these data, assumes there will be a uniform intercorrelation between outcome measures in the population. This is likely to be unrealistic. Nevertheless, further simulations showed that values for MEff are reasonably consistent for different correlation matrices that all have the same average off-diagonal correlation. Consider, for instance, the correlations between 4 variables shown in Figure 1 for Model L2. Within the blocks V1-V2 and V3-V4 the intercorrelation is *r,* but between blocks the intercorrelation is zero. There are six off-diagonal correlations and the mean off-diagonal is (2 * *r*/6). For instance, if *r* equals .5, then the mean off-diagonal value is .167. To see how the MEff correction is affected by correlation structure, we can compare MEff for Model L2 with the MEff obtained in Model L1 with the same off-diagonal correlation. This exercise shows that they are similar, as shown in Table 3.

**Table 3.** AlphaMEff values for Model L2 (odd rows) and Model L1 (even rows), with same mean off diagonal *r*. For Model L2, “Start *r*” is the value for nonzero off-diagonal correlations.

Start *r*	Model	Mean offdiag *r*	Alpha.MEff.4	Alpha.MEff.6	Alpha.MEff.8
0.2	L2	0.086	0.013	0.008	0.006
	L1	0.086	0.013	0.008	0.006
0.3	L2	0.129	0.013	0.009	0.006
	L1	0.129	0.013	0.008	0.006
0.4	L2	0.171	0.013	0.009	0.007
	L1	0.171	0.013	0.009	0.006
0.5	L2	0.214	0.013	0.009	0.007
	L1	0.214	0.013	0.009	0.007
0.6	L2	0.257	0.014	0.009	0.007
	L1	0.257	0.013	0.009	0.007
0.7	L2	0.300	0.014	0.010	0.008
	L1	0.300	0.013	0.009	0.007
0.8	L2	0.343	0.015	0.011	0.008
	L1	0.343	0.013	0.009	0.007

In other words, if estimating MEff from existing data, it is reasonable to base the estimate on the average off-diagonal correlation, regardless of whether the pattern of intercorrelations is uniform.

### Examples of application to published studies

Use of the lookup Table 2 can be illustrated with data from a study by Burgoyne et al. (2012), which evaluated a reading and language intervention for children with Down syndrome. A large number of assessments was carried out over various time points, but our focus here is on the five outcome measures that had been designated as “primary”, as they were “proximal to the content of the intervention”, i.e., they measured skills and knowledge that had been explicitly taught. The p-values reported by the authors (see Table 4) come from analyses of covariance comparing differences between intervention and control groups after 20 weeks of intervention, controlling for baseline performance, age and gender.

**Table 4.** P-values from Burgoyne *et al.* (2012). Bonferroni and MEff alpha for 6 variables with mean correlation of .6.

Measure	Reported p.value	Bonferroni: alpha = .01	MEff: alpha = .014
Letter-Sound knowledge	0.002	*	*
Phoneme blending	0.022		
Single word reading	0.002	*	*
Taught expressive Vocabulary	0.011		*
Taught receptive Vocabulary	0.062		

Whereas the Bonferroni-corrected alpha can be computed simply from knowledge of the number of outcome measures, the MEff-corrected alpha requires knowledge of the mean correlation between the outcome measures. In this case, this could be computed, ( *r* = .581), as the data were available in a repository ( Burgoyne et al., 2016). From Table 2, we see that with five outcome measures and *r* = .6, the adjusted alpha is .014. In this example, three outcomes have p-values below the critical alpha when MEff is used. If the more stringent Bonferroni correction is applied, only two outcomes achieve significance.

In this example the intercorrelation between outcome measures could be computed from deposited raw data; if these are not available, then it may still be possible to obtain plausible estimates of intercorrelation between outcome measures, especially if widely-used instruments are used. An example is provided by two randomized controlled trials of a memory training programme for children, Cogmed. In both studies, the Automated Working Memory Assessment battery ( Alloway, 2007) was used to assess outcome. Chacko et al. (2014) used four subtests, Dot Matrix, Spatial Recall, Digit Recall, and Listening Recall, and applied the Sidak-Bonferroni correction, with effective alpha of .013. The raw data are not available, but the test manual indicates that intercorrelations between these four measures range from .70 to .78. Thus we can use the lookup table (Table 2), which shows that with four variables with intercorrelation of .7, an effective alpha of .02 can be used. In practice this did not affect the interpretation of results, because two of the measures, Dot Matrix and Digit Recall, had associated p-values of < .001 and .005 respectively. The p-values for Spatial Recall and Listening Recall were .048 and .728 respectively, and so would not meet criteria for significance with MEff or Bonferroni methods.

The other study by Roberts et al. (2016) used a different subset of subtests from the same battery: Dot Matrix, Digit Recall, Backward Digit Recall, and Mister X, given at 6 months, 12 months and 24 months post-intervention. According to the test manual, intercorrelations between these subtests range from .65 to .80. These authors did not apply a correction for multiple comparisons. If Bonferroni correction had been used this would have given an alpha level of .004 (.05/12). The test manual indicates that test-retest reliability of the subscales ranges from .84 to .89. Thus overall, we can estimate the off-diagonal correlations for all 12 measures to be around .8, which the lookup table shows as corresponding to an effective alpha of .01. In this study, only the Dot Matrix task effect was significant after correction for multiple comparisons, with p < .001 at both 6 months and 12 months, but p = .14 at 24 months. Backward Digit Recall gave p = .04 at 6 months only, which would be nonsignificant if any correction for multiple comparisons were used. All other comparisons were null. In the next section, the implications of these findings for choosing methods is discussed further.

## Discussion

Some interventions are expected to affect a range of related processes. In such cases, the need to specify a single primary outcome tends to create difficulties, because it is often unclear which of a suite of outcomes is likely to show an effect. Note that the MEff approach does not give the researcher free rein to engage in p-hacking: the larger the suite of measures included in the study, the lower the adjusted alpha will be. It does, however, remove the need to pre-specify one measure as the primary outcome, when there is genuine uncertainty about which measure might be most sensitive to intervention.

A second advantage is that in effect, by including multiple outcome measures, one can improve the efficiency of a study, in terms of the trade-off between power and familywise errors. A set of outcome measures may be regarded as imperfect proxy indicators of an underlying latent construct, so we are in effect building in a degree of within-study replication by including more than one outcome measure.

The simulations showed that PCA gives higher power than MEff in the case where all outcomes are indicators of a single underlying factor. PCA, however, needs to be computed from raw data and so is not feasible when re-evaluating published studies, whereas MEff is feasible so long as the average off-diagonal correlation between outcomes can be estimated. PCA is also less powerful when the outcomes tap into heterogeneous constructs and do not load on one major latent factor. Some examples are provided where prior literature gives plausible estimates of intercorrelations between outcome measures. Of course, such estimates are never as accurate as the actual correlations from the reported data, which may vary depending on sample characteristics. Wherever possible, it is preferable to work with original raw data. However, where correlations are available from test manuals, or where previous studies have reported correlations between outcomes, then the researcher can consider how interpretation of results may be affected by assuming a given degree of dependency between outcome measures.

A possible disadvantage of using MEff or Bonferroni correction over PCA is that such approaches are likely to tempt researchers to interpret specific outcomes that fall below the revised alpha threshold as meaningful. They may be, of course, but when we create a suite of outcomes that differ only by chance, it is common for only a subset of them to reach the significance criterion. Any recommendation to use MEff should be accompanied by a warning that if a subset of outcomes shows an effect of intervention, this could be due to chance. It would be necessary to run a replication to have confidence in a particular pattern of results.

In this regard, the example of studies using the Automated Working Memory Assessment to evaluate intervention for children with memory and attentional difficulties ( Chacko et al., 2014; Roberts et al., 2016) are instructive. As reported in the test manual ( Alloway, 2007), intercorrelations between the subtests are high, supporting the idea of a general working memory factor that influences performance on all such measures. On that basis, it might seem preferable to reduce subtest scores to one outcome measure - either by using data reduction such as principal component analysis, or by using the method advocated in the test manual to derive a composite score. We know this is associated with an increase in reliability of measurement and statistical power. However, the results of the two studies sound a note of caution: in both trials there were large improvements in one subtest, Dot Matrix, at least in the short-term, while other measures did not show consistent gains. This kind of result has been much discussed in evaluations of computerised training, where it has been noted that one may see improvements in tasks that resemble the training exercises, ‘near transfer’, without any generalisation to other measures, ‘far transfer’ ( Aksayli, Sala, & Gobet, 2019). The very fact that measures are usually intercorrelated provides the rationale for hoping that training one skill will have an effect that generalises to other skills, and to everyday life. Yet, the verdict on this kind of training is stark: after much early optimism, working memory training leads to improvements on what was trained, but these do not extend to other areas of cognition. This shows us that careful thought needs to be given to the logic of how a set of outcome measures is conceptualised: should we treat them as interchangeable indicators of a single underlying factor, or are there reasons to expect that the intervention will have a selective impact on a subset of measures? Even when variables are intercorrelated in the general population, they may respond differently to intervention.

It is also worth noting that results obtained with the MEff approach will depend on assumptions embodied in the simulation that is used to derive predictions. Outcome measures simulated here are normally distributed, and uniform in their covariance structure. It would be of interest to evaluate MEff in datasets with different variable types, such as those used by Vickerstaff et al. (2021) that included binary as well as continuous data, as well as modeling the impact of missing data.

In sum, a recommendation against using multiple outcomes in intervention studies does not lead to optimal study design. Inclusion of several related outcomes can increase statistical power, without increasing the false positive rate, provided appropriate correction is made for the multiple testing. Compared to most other approaches for correlated outcomes, MEff is relatively simple. It could potentially be used to reevaluate published studies that report multiple outcomes but may not have been analysed optimally, provided we have some information on the average correlation between outcome measures.

## Data availability

### Underlying data

OSF: Revised ‘multiple outcomes’ using MEff, < https://doi.org/10.17605/OSF.IO/6GNB4> ( Bishop, 2022).

This project contains the following underlying data:

• Simulated raw data from 2000 runs for models L1, L2 and L3 (corresponding to L1, L2 and L2x respectively).

### Extended data

OSF: Revised ‘multiple outcomes’ using MEff, < https://doi.org/10.17605/OSF.IO/6GNB4> ( Bishop, 2022).

This project contains the scripts to generate and analyse simulated data. Two scripts are included:

1. Data_simulation_modelL.Rmd, which generates the simulated data under Data, computes power tables and creates plots for Figures 2-4.
2. Multiple_outcomes_revised.Rmd, which generates the text for the current article.

Data are available under the terms of the Creative Commons Zero “No rights reserved” data waiver (CC0 1.0 Public domain dedication).

## References

[ref14] Aksayli ND Sala G Gobet F : The cognitive and academic benefits of Cogmed: A meta-analysis. * Educational Research Review. * 2019;27:229–243. 10.1016/j.edurev.2019.04.003

[ref15] Alloway TP : *Automated Working Memory Assessment Manual.* London: Pearson Assessment;2007. https://www.R-project.org/

[ref1] Bishop DVM : Using multiple outcomes in intervention studies for improved trade-off between power and type I errors: The Adjust NVar approach [version 1; peer review: 2 not approved]. *F1000Research. * 2021;10:991. 10.12688/f1000research.73520.1 36925625 PMC10011751

[ref2] Bishop DVM : Revised ‘Multiple Outcomes’ Using MEff. OSF.[Dataset.]2022November 18. 10.17605/OSF.IO/6JF9T

[ref3] Burgoyne K Duff FJ Clarke PJ : Efficacy of a reading and language intervention for children with Down syndrome: A randomized controlled trial. * Journal of Child Psychology and Psychiatry, and Allied Disciplines. * 2012;53(10):1044–1053. 10.1111/j.1469-7610.2012.02557.x 22533801 PMC3470928

[ref4] Burgoyne K Duff FJ Clarke PJ : Reading and language intervention for children with Down syndrome. * Experimental data [data collection]. * 2016. 10.5255/UKDA-SN-852291

[ref16] Chacko A Bedard AC Marks DJ : A randomized clinical trial of Cogmed Working Memory Training in school-age children with ADHD: A replication in a diverse sample using a control condition. * Journal of Child Psychology and Psychiatry. * 2014;55(3):247–255. 10.1111/jcpp.12146 24117656 PMC3944087

[ref5] Cheverud JM : A simple correction for multiple comparisons in interval mapping genome scans. * Heredity. * 2001:87(1): Article 1. 10.1046/j.1365-2540.2001.00901.x 11678987

[ref6] Derringer J : A simple correction for non-independent tests. * PsyArXiv. * 2018. 10.31234/osf.io/f2tyw

[ref7] Lakens D : The 20% Statistician: One-sided tests: Efficient and Underused. *The 20% Statistician.* 2016, March 17. http://daniellakens.blogspot.com/2016/03/one-sided-tests-efficient-and-underused.html

[ref8] Moher D Hopewell S Schulz KF : CONSORT 2010 explanation and elaboration: Updated guidelines for reporting parallel group randomised trials. * BMJ (Clinical Research Ed.) * 2010;340: c869. 10.1136/bmj.c869 PMC2844943 20332511

[ref9] Nyholt DR : A simple correction for multiple testing for single-nucleotide polymorphisms in linkage disequilibrium with each other. *American Journal of Human Genetics.* 2004;74(4):765–769. 14997420 10.1086/383251 PMC1181954

[ref10] R Core Team : *R: A language and environment for statistical computing.* Vienna, Austria: R Foundation for Statistical Computing;2020. https://www.R-project.org/

[ref17] Roberts G Quach J Spencer-Smith M : Academic outcomes 2 years after working memory training for children with low working memory: A randomized clinical trial. * JAMA Pediatrics. * 2016;170(5): e154568. 10.1001/jamapediatrics.2015.4568 26954779

[ref11] Sainani K : Peer Review Report For: Using multiple outcomes in intervention studies for improved trade-off between power and type I errors: The Adjust NVar approach [version 1; peer review: 2 not approved]. * F1000Research. * 2021;10:991. 10.5256/f1000research.77175.r96192 36925625

[ref12] Vickerstaff V Ambler G King M : Are multiple primary outcomes analysed appropriately in randomised controlled trials? * A review. Contemporary Clinical Trials. * 2015;45(Pt A):8–12. 10.1016/j.cct.2015.07.016 26215934

[ref13] Vickerstaff V Ambler G Omar RZ : A comparison of methods for analysing multiple outcome measures in randomised controlled trials using a simulation study. * Biometrical Journal. Biometrische Zeitschrift. * 2021;63(3):599–615. 10.1002/bimj.201900040 33314364 PMC7984364

