# Supplementary material for: Using multiple outcomes in intervention studies: improving power while controlling type I errors
Source: F1000Res. 2023 Nov 6;10:991. Originally published 2021 Sep 30. [Version 3] doi: 10.12688/f1000research.73520.3 (PMC10011751; doi:10.12688/f1000research.73520.3)
Supplement: Peer review history

# Reviewer report: Reviewer response for version 3

**DOI:** 10.5256/f1000research.158245.r221079

**Published:** 2023-11-14

**Authors:** Daniel Lakens

**Affiliations:** 1 Human-Technology Interaction Group, Department of Industrial Engineering and Innovation Sciences, Eindhoven University of Technology, Eindhoven, The Netherlands

I already approved the report, and the new changes are small improvements that do not change my evaluation.

Is the rationale for developing the new method (or application) clearly explained?

Partly

Is the description of the method technically sound?

Yes

Are the conclusions about the method and its performance adequately supported by the findings presented in the article?

No

If any results are presented, are all the source data underlying the results available to ensure full reproducibility?

Yes

Are sufficient details provided to allow replication of the method development and its use by others?

Yes

Reviewer Expertise:

Applied statistics

I confirm that I have read this submission and believe that I have an appropriate level of expertise to confirm that it is of an acceptable scientific standard.

# Reviewer report: Reviewer response for version 2

**DOI:** 10.5256/f1000research.141308.r160158

**Published:** 2023-03-13

**Authors:** Kristin Sainani

**Affiliations:** 1 Department of Epidemiology and Population Health, Stanford University, Stanford, CA, USA

The focus of this paper has shifted from the original version. It now focuses on the Meff approach rather than the original proposed MinNVar approach. The goals of the paper have also shifted: (1) to identify situations in which the use of multiple primary outcomes with appropriate adjustment for multiple comparisons yields higher statistical power than a single primary outcome; and (2) to provide tools for re-evaluating already published papers that used multiple primary outcomes but failed to adjust for multiplicity.

 In shifting the focus of the paper, the authors have addressed my original concerns. I like the Meff approach because it is relatively straightforward and intuitive. So, I’m glad that this revised version provides a brief tutorial on Meff for psychologists. Table 1 also provides a nice intuitive illustration of how Meff works. I also appreciate that this new draft explores different possible patterns of correlations that reflect different underlying latent variables. This paints a more realistic picture and brings out some of the tradeoffs of the different approaches (PCA, Bonferroni, Meff).

 I think this paper accomplishes its stated goals, and is a useful resource. I have spot-checked a few of the simulations and see similar patterns to what the paper reports. I appreciate that the authors have made their code and data available.

 I have just a few minor suggestions:

1. Figures 2-4. I would recommend removing effect size=0.8. Effect size makes some difference but appears less important than number of outcomes and correlation strength. Furthermore, power is always high with effect size=0.8 and n=50, so it doesn’t add much information to display effect size=0.8. I also had trouble distinguishing the two dashed lines. Altogether, effect size=0.8 just makes the graph harder to read without adding a lot of extra information.   
2. It’s somewhat contradictory to say that the lookup tables are useful when you don’t have access to the underlying data but then to present an illustrative example for which the underlying data were available (Burgyone 2012). Presumably, if we had access to the full data, we could calculate Meff exactly (or account for multiple testing using other approaches). Are there any examples you could present where the data are not available but there is some way to roughly estimate the correlations? E.g., because of summary data in the paper or because the correlations can be roughly estimated from previous work using the same variables? This might be closer to the real-world use case for the lookup tables.

Is the rationale for developing the new method (or application) clearly explained?

Partly

Is the description of the method technically sound?

Partly

Are the conclusions about the method and its performance adequately supported by the findings presented in the article?

Partly

If any results are presented, are all the source data underlying the results available to ensure full reproducibility?

Yes

Are sufficient details provided to allow replication of the method development and its use by others?

Yes

Reviewer Expertise:

Statistics, Sports Medicine

I confirm that I have read this submission and believe that I have an appropriate level of expertise to confirm that it is of an acceptable scientific standard.

# Response

**Published:** 2023-10-28

**Authors:** Dorothy Bishop

**Affiliations:** University of Oxford, UK

Many thanks for the helpful recommendations. 

 Figures 2 - 4 have been revised in line with suggestions.  The original figures with effect size of .8 are still available on OSF, and a Wiki to that page on OSF has been added to explain the difference. 

 I have also added two more real-world examples, both dealing with evaluation of a memory intervention, Cogmed, for children.  It was possible to use the MEff lookup table because both studies used a published working memory battery, where correlations between the subscales can be found in the test manual. Although in practice this had little impact on the interpretation of these studies, it did show how alpha depends on the correction used:  one study had used Bonferroni correction, which was over-stringent, and the other had used no correction for multiple contrasts.  I found that this exercise not only provided an illustrative example of use of MEff, but it further emphasised the need to consider the underlying relationships between intervention and outcomes when deciding on an analytic strategy, and so I added a further comment about that in the Discussion.

# Reviewer report: Reviewer response for version 2

**DOI:** 10.5256/f1000research.141308.r160159

**Published:** 2023-02-21

**Authors:** Daniel Lakens

**Affiliations:** 1 Human-Technology Interaction Group, Department of Industrial Engineering and Innovation Sciences, Eindhoven University of Technology, Eindhoven, The Netherlands

This is an interesting revision, because the author has largely abandoned the original proposal, and switched to a different approach to control error rates when multiple dependent hypotheses are tested. As the goal of the paper is a practical tutorial, I think this switch is a valid and good choice. It also means many of my original comments are no longer relevant.

 The idea of creating lookup tables is a nice contribution. The biggest weakness is that correlations 1) are often unknown when data is not shared, and 2) are likely more varied than in the simulations. The authors admit this “In practice, the problem for the researcher is to estimate the intercorrelation between outcome measures if this is not known.”. They then give an applied example where the data was shared in a repository. What is missing is a clear instruction what to do when data is not shared in a repository. I would assume this then means 1) asking for the data, 2) if data is not provided making an informed guess, and 3) be careful in the interpretation, or performing some sensitivity analyses (e.g., X findings are significant, assuming correlations are not lower than Y). I think presenting a plan for when data is not available is a useful addition.

 I also still wonder what happens if there is substantial variation in the off-diagonal correlations. Maybe the authors feel this is rare in realistic datasets – then that could be mentioned.

 Minor comment

 The heading “The case against multiple outcomes” might confuse readers a bit, as you are arguing FOR multiple outcomes. So, maybe replace it by something like ‘Evaluating error rates for multiple outcomes’.

 I checked the manuscript for reproducibility, and reproduced the figures and data. I performed the simulations with a larger N, as I thought the patterns in the figures showed some surprising patterns (e.g., not purely decreasing or increasing) but found the same with a larger number of simulations, so that is not the issue.

Is the rationale for developing the new method (or application) clearly explained?

Partly

Is the description of the method technically sound?

Yes

Are the conclusions about the method and its performance adequately supported by the findings presented in the article?

No

If any results are presented, are all the source data underlying the results available to ensure full reproducibility?

Yes

Are sufficient details provided to allow replication of the method development and its use by others?

Yes

Reviewer Expertise:

Applied statistics

I confirm that I have read this submission and believe that I have an appropriate level of expertise to confirm that it is of an acceptable scientific standard.

# Response

**Published:** 2023-02-24

**Authors:** Dorothy Bishop

**Affiliations:** University of Oxford, UK

Thanks to the reviewer for the new evaluation.

 I’ll defer making modifications to the document until a second review is available, but just note a couple of points.

*What is missing is a clear instruction what to do when data is not shared in a repository. I would assume this then means 1) asking for the data, 2) if data is not provided making an informed guess, and 3) be careful in the interpretation, or performing some sensitivity analyses (e.g., X findings are significant, assuming correlations are not lower than Y). I think presenting a plan for when data is not available is a useful addition.*

 Response: I like this suggestion for a plan of action. A further step between 1 and 2 would be to do a search for other datasets using the same variables, which may give an indication of the range of expected correlation between them – while recognising that observed values may be influenced by factors such as range. So the “informed guess” would be informed by prior literature, if available.

*I also still wonder what happens if there is substantial variation in the off-diagonal correlations. Maybe the authors feel this is rare in realistic datasets – then that could be mentioned.*

 Response: On the contrary, I suspect variation in off-diagonal correlations is more common than uniformity. But in effect the most extreme version of this case is already modelled with model L2. In this models, the off-diagonal values are either zero or a specific value, r.max.  The appropriate comparison for these models is a model, L1, where the correlations are uniform, with r.avg equivalent to the average off-diagonal value. Consider the case in the bottom row of table 3. For model L2, we have r values of either 0 or .8.  For model L1, we have a corresponding value of r.avg of .343. The alpha values for L1 and L2 are in adjacent rows of table 3, and it is clear they differ only slightly. If you introduced more variability in model L2, with some r-values being intermediate between 0 and r.max, the difference between models L1 and L2 would be smaller.

# Response

**Published:** 2023-10-28

**Authors:** Dorothy Bishop

**Affiliations:** University of Oxford, UK

Thanks for your careful reading of the paper. I particularly appreciate you checking the reproducibility of the simulations; I realise this takes some work but it is good to have the reassurance that the patterns of results are reproducible.   

 Following suggestions by reviewer 1, I've added two examples of real-world studies where data are not available.  In the field of educational/psychological interventions, it can be possible to get estimates of intercorrelations between measures if well-established measures are used, as is the case in this example.  I've also added some thoughts on what to do when this is not the case, as you proposed. 

 Regarding the case of substantial variation in off-diagonal correlations, having played around with various scenarios, I think it's reasonable to regard model L2 as corresponding to that, because it has a mixture on the off-diagonal of variables with correlation of zero, and those with correlation of whatever is the maximum value for correlated measures from one factor.  For instance, if you look at the bottom row of Table 3, L2 is the case where the off-diagonal contains a mixture of r values of 0 and .8, and L1 is the case when the off-diagonals are uniform (and equivalent to the mean of L2 values). Yet the MEff varies only slightly.  I think that is about the most extreme variation you could get for off-diagonal values.

# Reviewer report: Reviewer response for version 1

**DOI:** 10.5256/f1000research.77175.r97181

**Published:** 2021-11-10

**Authors:** Daniel Lakens

**Affiliations:** 1 Human-Technology Interaction Group, Department of Industrial Engineering and Innovation Sciences, Eindhoven University of Technology, Eindhoven, The Netherlands

The author discusses more optimal ways to control error rates than the Bonferroni correction when researchers use multiple measures in a study that are positively correlated. In these cases the authors proposed to specify the number of variables that should be significant at the default alpha level (e.g., 0.05) to make sure the overall Type 1 error rate does not exceed 0.05.

 The difficulty with correcting for multiple comparisons based on the number of variables that are significant is that you want to prevent a situation where a researcher performs a Stroop effect test, a Simon effect test, and a test for precognition, and conclude the hypothesis is supported because 2 out of 3 tests are significant. The solution the author proposes is to only use this approach if 1) the same latent variable is measured in different measures, and 2) the correlation between these variables can not be zero. I believe this is a valid approach. These assumptions are discussed enough in the article, but on page 11 (of the pdf version), one might want to discuss that *whether* different measures actually are ‘replicants’ could be a matter of debate among peers. One could imagine a situation where researchers in a field who disagree about measures would also disagree about whether different measures are replicants.

 The comparison against the Bonferroni correction is one interesting baseline, but there is a large literature on how to correct for multiple comparisons that is also much more efficient than a Bonferroni correction, and which is the more interesting comparison. A strength of the Bonferroni correction is that it makes no assumptions about the variables that are corrected. But if one is willing to make assumptions, most importantly about the correlation between variables, more efficient approaches are available. How does this approach compare against other correction approaches? Although there are many correction approaches (this seems to be a particularly active field in neuroscience, where multiple comparisons when analyzing brain activation are common, and measures are strongly correlated), the following two references provide a starting point.

 Fan, J., Han, X., & Gu, W. (2012). Estimating False Discovery Proportion Under Arbitrary Covariance Dependence. *Journal of the American Statistical Association*, *107*(499), 1019–1035 ^1^

 Yekutieli, D., & Benjamini, Y. (1999). Resampling-based false discovery rate controlling multiple test procedures for correlated test statistics. *Journal of Statistical Planning and Inference*, *82*(1), 171–196 ^2^

 There are several things to consider. First, the proposed simulation based approach fixes the alpha level, and selects the number of variables that need to be significant. Numbers of variables are relatively crude, in that we can pick 1, 2, 3, 4, etc, but not 1.23 variables. Alternative approaches in the literature lower the alpha level. A benefit of these approaches is that the alpha level can be set at any value (e.g., 0.0352) to exactly control the Type 1 error rate. The consequences of this are also clear when we look at the figures and compare the principle component approach with the Adjust NVar approach. The familywise error rate for the Adjust NVar approach is often well below 0.05, while it is controlled at 0.05 in the principle component approach (and it is controlled at 0.05 in other approaches in the literature that lower the alpha level). The author discusses this (e.g., page 7 of the pdf version) in some detail, but the author seems slightly biased towards their own approach, stating that “the tradeoff between power and familywise error (expressed as a ratio) is higher for Adjust NVar.” It is not clear this ratio is a fair evaluation of the methods, and the information is difficult to distill from the figures (a Table with Type 1 error rates and Type 2 error rates would be more useful for this). This ratio is not so easy to summarize in a single sentence, I feel. If a design has 99.9% power, lowering the alpha from 0.05 to 0.02 has little effect on power, but if power is 0.8, lowering the alpha has a greater effect. Typically, the evaluation is done on the required sample size – which type of correction would require the smallest sample size? And then it becomes important to take a long more modern corrections for correlated variables as well.

 I could imagine other modern corrections are a bit more efficient, but the author makes a good point that the simplicity of the methods allows one to evaluate published studies. This might be a useful application. However, then I would like to urge the author to add an example. It would be good to see how this approach should be applied in a real use case (e.g., read a justification for why the measures are measuring the same latent construct, and how to make an assumption about the correlation) and how to interpret the results, depending on how many tests are significant.

 The current simulation is somewhat limited. The idea that all correlations are identical will not be true in practice, and this will complicate the application of the proposed technique. What is the recommendation in practice? Should authors choose the largest correlation or the smallest correlation? Which approach is more or less conservative? What is the effect of differences in standard deviations, and does it matter if measures with low correlations have larger standard deviations? I am not sure all these factors will have a large influence but users will most likely need to know what to do.

 It was very nice to see the paper was written in Rmarkdown. I was able to reproduce the results computationally (I did not repeat the simulations). As a minor comment, skipsim was no longer on line 207 but on 222. The files would be clearer if all simulation code was a separate R script that is run to generate the simulation data. The datafiles generated can then be read in at the top of the Rmd file. The ‘skipsim’ workaround does not improve clarity, and I had a hard time figuring out where the code reads in the data. In row 561, for example, the toybit file is written, but if I read it in, this is not needed. Separating the creation of data, and reading in data, makes this cleaner. I needed to create an ‘Images” folder to run the plot code on line 679 – this folder could be uploaded to the github repo perhaps?

 To conclude, I believe this current version of the manuscript needs some additional work, which could include a more extensive discussion of other corrections in the literature, an exploration of additional simulations, and a practical example of how to use this approach when analyzing published studies.

Is the rationale for developing the new method (or application) clearly explained?

Partly

Is the description of the method technically sound?

Yes

Are the conclusions about the method and its performance adequately supported by the findings presented in the article?

No

If any results are presented, are all the source data underlying the results available to ensure full reproducibility?

Yes

Are sufficient details provided to allow replication of the method development and its use by others?

Yes

Reviewer Expertise:

Applied statistics

I confirm that I have read this submission and believe that I have an appropriate level of expertise to state that I do not consider it to be of an acceptable scientific standard, for reasons outlined above.

## References

[1] : Estimating False Discovery Proportion Under Arbitrary Covariance Dependence. *J Am Stat Assoc* .2012;107(499) : 10.1080/01621459.2012.720478 1019-1035 10.1080/01621459.2012.720478 24729644PMC3983872

[2] : Resampling-based false discovery rate controlling multiple test procedures for correlated test statistics. *Journal of Statistical Planning and Inference* .1999;82(1-2) : 10.1016/S0378-3758(99)00041-5 171-196 10.1016/S0378-3758(99)00041-5

# Response

**Published:** 2022-11-18

**Authors:** Dorothy Bishop

**Affiliations:** University of Oxford, UK

I thank the reviewer for such a comprehensive and constructive review. The two reviews complemented one another very nicely, and helped me see how to restructure and revise the paper to make it clearer.  

 As background, I should explain that this paper grew out of an attempt to write a primer on how to analyse/evaluate published intervention studies for students/professionals in allied health professions/education. My concern was that Bonferroni correction is often too conservative, but explanations of other methods are typically highly complex, so I was looking for a simpler rule of thumb that could be useful in interpreting published studies. I have emphasised this rationale more in the revised paper. 

**Response to specific points.**

 1. The difficulty with correcting for multiple comparisons based on the number of variables that aresignificant is that you want to prevent a situation where a researcher performs a Stroop effect test, a Simon effect test, and a test for precognition, and conclude the hypothesis is supported because 2 out of 3 tests are significant. The solution the author proposes is to only use this approach if 1) the same latent variable is measured in different measures, and 2) the correlation between these variables can not be zero. I believe this is a valid approach. These assumptions are discussed enough in the article, but on page 11 (of the pdf version), one might want to discuss that *whether* different measures actually are ‘replicants’ could be a matter of debate among peers. One could imagine a situation where researchers in a field who disagree about measures would also disagree about whether different measures are replicants

*Response: This is an important point, which is similar to issues raised by reviewer 1, so I have now extended the simulations to take into account situations where outcomes may not all be indicators of one factor.*

 2. Alternative methods for correction for multiple comparisons.

*Response: The reviewer recommends two references with alternative approaches to multiple comparison correction. As I noted above, one goal was to keep things simple: so I am reluctant to do a full comparison of all methods, especially since it is clear that I need to consider different underlying data structures.  I like the relative simplicity of the MEff approach recommended by reviewer 1, and so I am now presenting a comparison of Bonferroni, PCA and MEff.*

 3. I could imagine other modern corrections are a bit more efficient, but the author makes a good point that the simplicity of the methods allows one to evaluate published studies. This might be a useful application. However, then I would like to urge the author to add an example. It would be good to see how this approach should be applied in a real use case (e.g., read a justification for why the measures are measuring the same latent construct, and how to make an assumption about the correlation) and how to interpret the results, depending on how many tests are significant.

*Response: Again, this converges nicely with the recommendations of reviewer 1, and I  have now restructured the paper to focus more on this aspect and to give a real-life example.*

 4. The current simulation is somewhat limited. The idea that all correlations are identical will not be true in practice, and this will complicate the application of the proposed technique. What is the recommendation in practice? Should authors choose the largest correlation or the smallest correlation? Which approach is more or less conservative? What is the effect of differences in standard deviations, and does it matter if measures with low correlations have larger standard deviations? I am not sure all these factors will have a large influence but users will most likely need to know what to do.

*Response: I now contrast three models with different patterns of intercorrelation between intervention and outcome. Potentially, there is no end to the options to consider, but I found it reassuring that with the MEff approach, the adjusted alpha was similar for different correlated variables, provided the mean off-diagonal correlation was constant.*

 5. It was very nice to see the paper was written in Rmarkdown. I was able to reproduce the results computationally (I did not repeat the simulations). As a minor comment, skipsim was no longer on line 207 but on 222. The files would be clearer if all simulation code was a separate R script that is run to generate the simulation data. The datafiles generated can then be read in at the top of the Rmd file. The ‘skipsim’ workaround does not improve clarity, and I had a hard time figuring out where the code reads in the data. In row 561, for example, the toybit file is written, but if I read it in, this is not needed. Separating the creation of data, and reading in data, makes this cleaner. I needed to create an ‘Images” folder to run the plot code on line 679 – this folder could be uploaded to the github repo perhaps?

*Response: I have now separated the simulation and generation of corresponding figures from the code to write the paper.*

# Reviewer report: Reviewer response for version 1

**DOI:** 10.5256/f1000research.77175.r96192

**Published:** 2021-10-12

**Authors:** Kristin Sainani

**Affiliations:** 1 Department of Epidemiology and Population Health, Stanford University, Stanford, CA, USA

The paper presents a method for controlling the familywise error rate when testing multiple outcomes: Adjust NVar. Unlike multiple testing adjustments that lower the p-value threshold, the idea behind Adjust NVar is to require a minimum number of p-values (MinNSig) to meet a nominal p<.05 threshold.

 When outcomes are independent, MinNSig is defined as follows, where M is the number of outcomes:

 X~binomial(M, 0.05)

 MinNSig is the minimum x for which P(X>=x)<.05 or, equivalently, P(X < x)>.95.

 For example, if M=6, then:

 P(X=0)=.95**6=0.735

 P(X<=1)=P(X<2)=0.735+6*.95**5*.05=.232+.735=.967

 Thus, MinNSig=2.

 When outcomes are correlated, there is no simple formula for obtaining MinNSig, so the author has used a simulation approach to account for varying correlation structures.

 The idea is novel and has several merits. In particular, the approach closely matches what many researchers already do in the published literature. Many published studies apply a p-value cutoff of 0.05 to multiple outcomes without correcting for multiple testing (or designating a primary outcome). I can envision the AdjustNVar approach being a useful heuristic for re-evaluating published studies that used multiple outcomes but failed to account for multiple testing in any way. For example, if an RCT reported 10 outcomes with only 1 significant result (p<.05), readers can easily recognize that this is compatible with a chance finding. But what if the trial found 2 significant results or 3? And what if the outcomes are moderately correlated instead of independent? I can envision researchers using a table such as Table 2 of the AdjustNVar paper to make a quick assessment based on the number of outcomes reported and a rough guess at the correlation structure.

 However, I am less convinced of the value of AdjustNVar as a formal tool for controlling the familywise error rate in a planned study. At a minimum, further development and a broader set of simulations would be required to support such a recommendation. The current manuscript describes three alternatives to specifying a single primary outcome in an RCT: (1) Bonferroni adjustment, (2) permutation tests, and (3) use of PCA to derive a single composite outcome. But this ignores existing p-value adjustment methods that are less conservative than Bonferroni. For example, the “M-effective” (Meff) approach accomplishes many of the same goals as AdjustNVar (see: Cheverud (2001) ^1^, Nyholt (2004) ^2^, and Derringer (2018) ^3^). In the Meff approach, one adjusts the p-value threshold by dividing by the effective number of outcomes (Meff) rather than the actual number of outcomes (M). Meff is based on the eigenvalues of the correlation matrix of the outcomes. Where Eigen is the observed vector of eigenvalues from the correlation matrix of the outcomes, Meff is calculated as:

 Meff = 1 + (M-1)*(1-(Var(Eigen)/M))

 Bonferroni threshold = alpha/M

 Meff threshold = alpha/Meff

 Like AdjustNVar, Meff is simple and accounts for correlated outcomes. But I believe it has several advantages over AdjustNVar: (1) Meff precisely controls the Type I error rate, whereas AdjustNVar has varying Type I error rates that cannot be precisely controlled by the investigator; (2) Meff accounts for the correlation structure observed in the data, whereas AdjustNVar requires the investigator to guess at the correlation structure; if this guess is far off (which could easily be the case), this would lead to poor Type I error control.

 This paper has numerous strengths, including the novelty of the idea; the potential use as a heuristic for re-interpreting flawed published papers; the concision of the writing; and the availability of all code and data. The major limitations of the paper are: (1) it presents an overly narrow set of simulations that do not capture most realistic situations, but then makes overly broad claims based on these simulations. (2) it does not compare AdjustNVar to existing approaches that are less conservative than Bonferroni, such as Meff and (3) it does not address the different reasons why researchers may be including multiple outcomes, but these different reasons lead to markedly different correlation structures.

 Specific comments:

1. The paper would benefit from further consideration of the reasons why researchers may include multiple outcomes. The paper focuses on the case where an intervention is “expected to affect a range of related processes.” The simulations make assumptions that match this case, assuming equal correlations across outcomes and equal true effects for each outcome. But researchers may include multiple outcomes for many other reasons, such as: (a) they aren’t sure which process the intervention will affect, (b) they believe the intervention may affect two different processes but they measure each process with several different measurements to “hedge their bets”, or (c) they include a “soft” endpoint in addition to a “hard” endpoint because the “hard” endpoint may occur too rarely. Each of these cases corresponds to different assumptions for the simulations. For example, (b) would be expected to have two clusters of highly correlated variables that are only weakly correlated with each, which will affect MinNSig.
2. The paper suggests that AdjustNVar could be used in study planning—researchers would guess at the correlation structure and set a MinNSig ahead of time. But if they guess the correlation structure incorrectly, such as underestimating the true correlation, then they may choose a MinNSig that does not adequately control Type I error.
3. The “quantum nature” of AdjustNVar is not a desirable characteristic. The researcher is unable to precisely control the Type I error rate. In planning a study in which the correlation is expected to be 0.4, for example, Table 2 would suggest that the researcher should then always choose 9 outcomes over 5-8 outcomes, since 9 maximizes the chances of getting at least 3 p-values <.05. This is one reason I prefer Meff, which precisely controls the Type I error rate.
4. This description is misleading: “Should we dismiss the trial as showing no benefit? We can use the binomial theorem to check the probability of obtaining this result if the null hypothesis is true and the measures are independent: it is 0.033, clearly below the 5% alpha level.” The description gives the misleading impression that one would be justified in re-evaluating a paper that used a Bonferroni correction by instead applying the criterion of at least two p-values <.05. But doing so would inflate the Type I error rate. Results would have been declared significant if EITHER at least one p-value met the Bonferroni threshold OR at least two p-values were <.05 — leading to an effective Type I error rate of 7% (assuming independent outcomes). Note that this example reappears in the discussion and also mistakenly implies that had three p-values been <.05, we would have been able to reject the null hypothesis. But this is not the case because the results were already subjected to Bonferroni, and additionally subjecting them to AdjustNVar makes the effective Type I error rate higher than 5%. AdjustNVar should be applied only to re-evaluate studies that failed to incorporate any adjustments for multiple testing originally.  
5. I found Table 1 confusing on first read, and I would recommend simplifying it by focusing on a single number of outcomes rather than both 2 outcomes and 4 outcomes and by removing discussions of ranking p-values. (The p-value ranking isn’t important — this is just part of the mechanics of how the algorithm is calculating MinNSig, so I don’t think it’s needed.) For example, you could just focus on calculating MinNSig for 6 outcomes. Show 6 columns of p-values for the 6 outcomes, and then show a single final column that tabulates the number of p-values <.05 for each simulated trial. Then show a frequency table of how many simulations out of 1000 resulted in 0 p-values <.05, 1 p-value <.05, 2 p-values <.05, etc. Then indicate that MinNSig occurs at one number above when the cumulative frequency crosses 95%.
6. I’m unclear as to why the paper focuses on one-tailed tests, which are less common in the literature.  I think it would be more useful to present two-tailed tests in Table 2 or to present two tables — one for one-tailed tests and one for two-tailed tests. This makes a difference in a few MinNSig values.
7. Figures 1-3: These figures compare AdjustNVar to a single study outcome. I think the logic behind this comparison is flawed, however. It is comparing apples to oranges. The simulation assumes that, when applying AdjustNVar, ALL variables studied have a true effect. This, in effect, stacks the deck on statistical power for *any* method that considers multiple outcomes rather than a single outcome. For example, I ran a simulation comparing Bonferroni with 6 outcomes compared to a single outcome with n=50 per group. When the correlation is <0.8, Bonferroni also has more power than the single outcome. And, when I compared the Meff strategy to a single outcome in a variety of scenarios, Meff was always more powerful than a single outcome. I think a more useful comparison would be to directly compare different methods that handle multiple outcomes (e.g., PC to AdjustNVar to Meff).
8. Related to comment (7), I don’t think the paper is justified in making this broad claim: “The Adjust NVar approach can achieve a more efficient trade-off between power and type I error rate than use of a single outcome when there are three or more moderately intercorrelated outcome variables.” This conclusion is true only when the intervention truly affects ALL outcomes, which is a narrow and arguably unrealistic case. A more realistic scenario is where the intervention works only on a subset of outcomes. In this case, the single variable strategy will be more statistically powerful than the multiple-variable strategies if you choose the right variable.
9. Figures 4-6. Same issue as for Figures 1-3: comparing the principal components composite variable strategy (PC) to a single outcome is flawed because the simulation “stacks the deck” for PC by assuming that all outcomes have a true effect. I believe that Figures 1-6 should focus instead on comparisons of different methods for handling multiple outcomes.
10. The article claims that power is only “slightly lower” for AdjustNVar compared with the PC strategy. However, when I run simulations comparing PC to AdjustNVar, I get consistently higher statistical power for PC and I would characterize the difference as more than just “slight”. For example, with N=50, global corr=0.6, global ES=0.3, and 6 outcomes (two-tailed test), I get power of 35% for AdjustNVar versus 37% for Meff versus 46% for PC.
11. PC is always more powerful than AdjustNVar and Meff when we assume that all outcomes have a true effect. However, PC is not more powerful when we assume that only a subset of outcomes have true effects. For example, if I tweak the above simulation so that only three outcomes out of six have true effects of 0.3, this changes the power to 15% for PC and AdjustNVar, and 28% for Meff. This illustrates why one narrow simulation is insufficient for making general conclusions about the tradeoffs in performance between the different methods.
12. In my simulations, Meff consistently has higher statistical power than AdjustNVar, which is a function of the fact that Meff always has a 5% Type I error rate whereas that of AdjustNVar is variable and sometimes lower than 5%. I don’t view it as a strength that AdjustNVar results in arbitrarily lower Type I error rates. It is better for the investigator to be able to precisely control the tradeoff between Type I and Type II error. Meff allows this whereas AdjustNVar does not.
13. Figures 1-6: I found these graphs hard to read as they don’t have a clear take-home point. I would suggest removing the single outcome comparisons altogether and then reformatting so that the graphs directly compare AdjustNVar to Meff and PC (three lines). In one graph, hold effect size, sample size, and N outcomes constant, and then show the power of the three methods as a function of increasing correlation. In another graph, hold correlation, sample size, and effect size constant, and show the power of the three methods as a function of N outcomes. And so on. All methods aim to control the familywise error rate at 5%, so this should not be a variable. The fact that AdjustNVar sometimes results in a lower Type I error rate is incidental — it arises as a quirk of the method not as the intent of the researcher.
14. Where AdjustNVar may have an advantage over a method like Meff is for re-evaluating flawed published studies where researchers used multiple outcomes but did not account in any way for multiple testing. An AdjustNVar table such as Table 2 would give readers a quick way to reevaluate such studies without the need for any calculations or access to raw data. I would focus the paper more on this application. 

Is the rationale for developing the new method (or application) clearly explained?

Partly

Is the description of the method technically sound?

Partly

Are the conclusions about the method and its performance adequately supported by the findings presented in the article?

Partly

If any results are presented, are all the source data underlying the results available to ensure full reproducibility?

Yes

Are sufficient details provided to allow replication of the method development and its use by others?

Yes

Reviewer Expertise:

Statistics, Sports Medicine

I confirm that I have read this submission and believe that I have an appropriate level of expertise to state that I do not consider it to be of an acceptable scientific standard, for reasons outlined above.

## References

[1] : A simple correction for multiple comparisons in interval mapping genome scans. *Heredity (Edinb)* .2001;87(Pt 1) : 10.1046/j.1365-2540.2001.00901.x 52-8 10.1046/j.1365-2540.2001.00901.x 11678987

[2] : A simple correction for multiple testing for single-nucleotide polymorphisms in linkage disequilibrium with each other. *Am J Hum Genet* .2004;74(4) : 10.1086/383251 765-9 10.1086/383251 14997420PMC1181954

[3] : A simple correction for non-independent tests.2018; 10.31234/osf.io/f2tyw 10.31234/osf.io/f2tyw

# Response

**Published:** 2022-11-18

**Authors:** Dorothy Bishop

**Affiliations:** University of Oxford, UK

Thanks for the very useful evaluation of this paper, which has prompted further thoughts and a revised manuscript. There was some convergence of views of the two reviewers, although the specific recommendations varied. I thank the reviewer for engaging so thoroughly with the manuscript and for helping improve it. 

 General point A: AdjustNVar as a heuristic. 

 Reviewer 1 writes: The idea is novel and has several merits. In particular, the approach closely matches what many researchers already do in the published literature. Many published studies apply a p-value cutoff of 0.05 to multiple outcomes without correcting for multiple testing (or designating a primary outcome). I can envision the AdjustNVar approach being a useful heuristic for re-evaluating published studies that used multiple outcomes but failed to account for multiple testing in any way. For example, if an RCT reported 10 outcomes with only 1 significant result (p<.05), readers can easily recognize that this is compatible with a chance finding. But what if the trial found 2 significant results or 3? And what if the outcomes are moderately correlated instead of independent? I can envision researchers using a table such as Table 2 of the AdjustNVar paper to make a quick assessment based on the number of outcomes reported and a rough guess at the correlation structure. 

*Response: After exploring Meff, I decided not to proceed with the AdjustNVar approach, as I think a lookup table derived from MEff would achieve the same effect but without the problems arising from the quantal nature of N variables.*

 General Point B: Need to consider MEff

 Reviewer 1 notes that the MEff approach accomplishes many of the same goals as AdjustNVar, and is preferable in many respects.  

*Response: I had been unaware of MEff, and having consulted the references, I agree it is an elegant solution to the problem I was trying to tackle that avoids some of the limitations of AdjustNvar, particularly the need to assume a given correlation structure. I initially thought this approach had only been used in genetics and not been applied in psychology, but the final reference pointed to the work of Derringer, who has done a great job in a preprint that provides a tutorial in its use. I don’t think it is much used in intervention research, which is the context I am particularly interested in, and so I think it is worthwhile updating the article so that it serves as a basic introduction to MEff, with discussion of factors affecting power.*

*Accordingly, I have changed the focus to compare different methods with a focus on MEff.*

 Specific comments:

 1. The paper would benefit from further consideration of the reasons why researchers may include multiple outcomes. The paper focuses on the case where an intervention is

 “expected to affect a range of related processes.” The simulations make assumptions that

 match this case, assuming equal correlations across outcomes and equal true effects for

 each outcome. But researchers may include multiple outcomes for many other reasons,

 such as: (a) they aren’t sure which process the intervention will affect, (b) they believe the

 intervention may affect two different processes but they measure each process with several

 different measurements to “hedge their bets”, or (c) they include a “soft” endpoint in

 addition to a “hard” endpoint because the “hard” endpoint may occur too rarely. Each of

 these cases corresponds to different assumptions for the simulations. For example, (b)

 would be expected to have two clusters of highly correlated variables that are only weakly

 correlated with each, which will affect MinNSig. 

*Response: This really got me thinking and I have now incorporated some further simulations where correlations are not uniform, as also added more discussion of this issue*

 2. The paper suggests that AdjustNVar could be used in study planning—researchers would

 guess at the correlation structure and set a MinNSig ahead of time. But if they guess the

 correlation structure incorrectly, such as underestimating the true correlation, then they

 may choose a MinNSig that does not adequately control Type I error. 

*Response: Agreed. This is now dropped.*

 3. The “quantum nature” of AdjustNVar is not a desirable characteristic. The researcher is

 unable to precisely control the Type I error rate. In planning a study in which the correlation

 is expected to be 0.4, for example, Table 2 would suggest that the researcher should then

 always choose 9 outcomes over 5-8 outcomes, since 9 maximizes the chances of getting at

 least 3 p-values <.05. This is one reason I prefer Meff, which precisely controls the Type I

 error rate. 

*Response: Agreed. AdjustNVar now dropped*

 4. This description is misleading: “Should we dismiss the trial as showing no benefit? We can

 use the binomial theorem to check the probability of obtaining this result if the null

 hypothesis is true and the measures are independent: it is 0.033, clearly below the 5% alpha

 level.” The description gives the misleading impression that one would be justified in reevaluating a paper that used a Bonferroni correction by instead applying the criterion of at

 least two p-values <.05. But doing so would inflate the Type I error rate. Results would have

 been declared significant if EITHER at least one p-value met the Bonferroni threshold OR at

 least two p-values were <.05 — leading to an effective Type I error rate of 7% (assuming

 independent outcomes). Note that this example reappears in the discussion and also

 mistakenly implies that had three p-values been <.05, we would have been able to reject the

 null hypothesis. But this is not the case because the results were already subjected to

 Bonferroni, and additionally subjecting them to AdjustNVar makes the effective Type I error

 rate higher than 5%. AdjustNVar should be applied only to re-evaluate studies that failed to

 incorporate any adjustments for multiple testing originally. 

*Response: thanks for this clarification. As AdjustNVar is now omitted, this no longer applies.*

 5. I found Table 1 confusing on first read, and I would recommend simplifying it by focusing

 on a single number of outcomes rather than both 2 outcomes and 4 outcomes and by

 removing discussions of ranking p-values. (The p-value ranking isn’t important — this is just

 part of the mechanics of how the algorithm is calculating MinNSig, so I don’t think it’s

 needed.) For example, you could just focus on calculating MinNSig for 6 outcomes. Show 6

 columns of p-values for the 6 outcomes, and then show a single final column that tabulates

 the number of p-values <.05 for each simulated trial. Then show a frequency table of how

 many simulations out of 1000 resulted in 0 p-values <.05, 1 p-value <.05, 2 p-values <.05, etc.

 Then indicate that MinNSig occurs at one number above when the cumulative frequency

 crosses 95%. 

*Response: this no longer applies as tables are redone*

 6. I’m unclear as to why the paper focuses on one-tailed tests, which are less common in the

 literature. I think it would be more useful to present two-tailed tests in Table 2 or to present

 two tables — one for one-tailed tests and one for two-tailed tests. This makes a difference in

 a few MinNSig values. 

*Response: One-tailed tests have a bad reputation because so often they are misused to just require a lower level of significance when there are no directional predictions, but there are contexts in which they are entirely appropriate, and that includes the kinds of intervention study that is the focus of attention here. You can reasonably predict that an intervention will improve performance rather than worsen it. Reviewer 2 wrote a blogpost about this which I find convincing: http://daniellakens.blogspot.com/2016/03/one-sided-tests-efficient-and-underused.html. I have now explained this further.*

 7. Figures 1-3: These figures compare AdjustNVar to a single study outcome. I think the logic

 behind this comparison is flawed, however. It is comparing apples to oranges. The

 simulation assumes that, when applying AdjustNVar, ALL variables studied have a true

 effect. This, in effect, stacks the deck on statistical power for *any* method that considers

 multiple outcomes rather than a single outcome. For example, I ran a simulation comparing

 Bonferroni with 6 outcomes compared to a single outcome with n=50 per group. When the

 correlation is <0.8, Bonferroni also has more power than the single outcome. And, when I

 compared the Meff strategy to a single outcome in a variety of scenarios, Meff was always

 more powerful than a single outcome. I think a more useful comparison would be to

 directly compare different methods that handle multiple outcomes (e.g., PC to AdjustNVar

 to Meff). 

*Response: Thanks again for helping clarify what is being simulated here. I hope this is clearer in the current article. The figures have been redrawn and I hope are now clearer. Perhaps one takeaway point is that, in being concerned to control type I error, the CONSORT recommendations appear to ignore the gains in power that can be achieved with multiple outcomes.*

 8. Related to comment (7), I don’t think the paper is justified in making this broad claim: “The

 Adjust NVar approach can achieve a more efficient trade-off between power and type I error

 rate than use of a single outcome when there are three or more moderately intercorrelated

 outcome variables.” This conclusion is true only when the intervention truly affects ALL

 outcomes, which is a narrow and arguably unrealistic case. A more realistic scenario is

 where the intervention works only on a subset of outcomes. In this case, the single variable

 strategy will be more statistically powerful than the multiple-variable strategies if you

 choose the right variable.

 Figures 4-6. Same issue as for Figures 1-3: comparing the principal components composite

 variable strategy (PC) to a single outcome is flawed because the simulation “stacks the deck”

 for PC by assuming that all outcomes have a true effect. I believe that Figures 1-6 should

 focus instead on comparisons of different methods for handling multiple outcomes.

*Response: same as for point 6; this is clearly a key issue, and I think it is clearer now that the alternative models (L2 and L2x) are included.*

 9. The article claims that power is only “slightly lower” for AdjustNVar compared with the PC

 strategy. However, when I run simulations comparing PC to AdjustNVar, I get consistently

 higher statistical power for PC and I would characterize the difference as more than just

 “slight”. For example, with N=50, global corr=0.6, global ES=0.3, and 6 outcomes (two-tailed

 test), I get power of 35% for AdjustNVar versus 37% for Meff versus 46% for PC. 

*Response: thanks for pushing back on this – this is fair comment.*

 10.PC is always more powerful than AdjustNVar and Meff when we assume that all outcomes

 have a true effect. However, PC is not more powerful when we assume that only a subset of

 outcomes have true effects. For example, if I tweak the above simulation so that only three

 outcomes out of six have true effects of 0.3, this changes the power to 15% for PC and

 AdjustNVar, and 28% for Meff. This illustrates why one narrow simulation is insufficient for

 making general conclusions about the tradeoffs in performance between the different

 methods. 

*Response: agreed.*

 11. In my simulations, Meff consistently has higher statistical power than AdjustNVar, which is a function of the fact that Meff always has a 5% Type I error rate whereas that of AdjustNVar

 is variable and sometimes lower than 5%. I don’t view it as a strength that AdjustNVar

 results in arbitrarily lower Type I error rates. It is better for the investigator to be able to

 precisely control the tradeoff between Type I and Type II error. Meff allows this whereas

 AdjustNVar does not. 

*Response: fair comment*

 12. Figures 1-6: I found these graphs hard to read as they don’t have a clear take-home point. I would suggest removing the single outcome comparisons altogether and then reformatting

 so that the graphs directly compare AdjustNVar to Meff and PC (three lines). In one graph,

 hold effect size, sample size, and N outcomes constant, and then show the power of the

 three methods as a function of increasing correlation. In another graph, hold correlation,

 sample size, and effect size constant, and show the power of the three methods as a

 function of N outcomes. And so on. All methods aim to control the familywise error rate at

 5%, so this should not be a variable. The fact that AdjustNVar sometimes results in a lower

 Type I error rate is incidental — it arises as a quirk of the method not as the intent of the

 researcher.

*Response: Agreed. Graphs reformatted as suggested and I hope are now much easier to interpret.*

 13. Where AdjustNVar may have an advantage over a method like Meff is for re-evaluating

 flawed published studies where researchers used multiple outcomes but did not account in

 any way for multiple testing. An AdjustNVar table such as Table 2 would give readers a quick

 way to reevaluate such studies without the need for any calculations or access to raw data. I

 would focus the paper more on this application. 

*Response: Agreed. The paper has been revised to make this point.*
